# Experimental and machine learning prediction of compressive strength of chemically activated RHA based RAC using SHAP and PDP analysis

**DOI:** 10.1038/s41598-025-25592-2

**Published:** 2025-12-04

**Authors:** Ahmed A. Alawi Al-Naghi, Tariq Ali, Inamullah Inam, Muhammad Zeeshan Qureshi, Muhammad Sarmad Mahmood, Nabil Ben Kahla, Nejib Ghazouani

**Affiliations:** 1 Civil Engineering Department, University of Ha’il, 55476 Ha’il, Saudi Arabia; 2 Department of Civil Engineering, Swedish College of Engineering and Technology, Wah, 47080 Pakistan; 3 Department of Civil Engineering, Engineering Faculty, Laghman University, Mehtarlam, Afghanistan; 4 Department of Civil Engineering, University of Engineering and Technology, Taxila, Pakistan; 5 Department of Civil Engineering, College of Engineering, King Khalid University, PO Box 394, 61411 Abha, Kingdom of Saudi Arabia; 6 Center for Engineering and Technology Innovations, King Khalid University, 61421 Abha, Saudi Arabia; 7 Mining Research Center, Northern Border University, 73213 Arar, Saudi Arabia

**Keywords:** Recycled concrete aggregate, Rice husk ash, Machine learning, Silica fume, Mechanical and durability properties, Civil engineering, Engineering

## Abstract

The increasing demand for sustainable construction materials necessitates the effective reuse of industrial and agricultural waste in high-performance concrete (HPC). However, challenges such as strength loss due to recycled concrete aggregates (RCA) and variable performance of supplementary cementitious materials hinder widespread adoption. This study addresses these challenges by investigating the synergistic effect of chemically activated rice husk ash (RHA), RCA (0–100%), and foundry sand on the compressive strength and durability of HPC. Six experimental groups were prepared: one with inactivated RHA and five with chemically activated RHA using 3.5% sodium sulfate (Na_2_SO_4_), combined with RCA replacement levels of 0%, 40%, 60%, 80%, and 100%. All mixes included 20% FS as partial fine aggregate replacement and constant silica fume. Compressive strength was measured at 3, 7, 14, 28, 56, 90, and 120 days, while durability was evaluated through acid exposure tests over 4 months. To complement the experimental study, machine learning models including K-Nearest Neighbors, Random Forest, Artificial Neural Networks, and Extreme Gradient Boosting were applied to predict compressive strength. Among them, XGB outperformed others with an R^2^ of 0.951, RMSE of 3.222 MPa, and MAE of 1.862 MPa. SHAP and Partial Dependence Plot (PDP) analyses revealed curing age, RCA, and Na_2_SO_4_ content as key influencing factors. This study concludes that up to 40% RCA can be effectively used in HPC with activated RHA and FS without compromising long-term strength and acid resistance. The integration of interpretable ML models with detailed experimental validation provides a robust framework for sustainable concrete design.

**Supplementary Information:**

The online version contains supplementary material available at 10.1038/s41598-025-25592-2.

## Introduction

The construction industry is developing rapidly, due to which the production of cement significantly increases, and has become a major factor leading to increasing carbon emissions and aggravating environmental pollution^1,2^. The cement industry accounts for about 8% of global carbon emissions^3^. The partial substitution of cement by low-energy industrial and agricultural solid waste can reduce the increase of carbon emissions, contributing to energy conservation and environmental protection^4–6^. Furthermore, the construction industry is one of the largest contributors to environmental pollution, that occurs during the construction process and after the service life of structures^7,8^. Thus, it is a key contributor to the global carbon footprint^9^. According to Anik et al.^10^ reports, the waste generated from the depletion of natural resources, the concrete industry accounts for approximately 50% of the total waste generated in construction. Arabani and Azarhoosh^11^ stated that concrete constitutes almost 75% of the waste produced at construction and demolition (C&D) sites worldwide and 70% of the waste from C&D sites globally. Thus, the concrete industry is one of the most unsustainable sectors as it disposes of its waste as landfill.

High-performance concrete (HPC) is characterized by superior mechanical properties, durability, and capacity to resist environmental and chemical attacks with respect to traditional concrete^12^. The inclusion of supplementary cementitious materials (SCMs) like micro silica, alkaline-activated rice husk ash, fly ash, and ground granulated blast furnace slag allows for a fine-grained matrix of cement, yielding significant enhancements in compressive strength and impermeability^13–17^. The properties of HPC made with Recycled Concrete Aggregate (RCA) depend on variables such as overall quality (replacement level) and optional presence of pozzolanic materials. The mortar content in RCA is an erroneous factor influencing the mechanical properties and rheological behaviors of HPC. Moreover, the value of water demand increases owing to the high porosity of RCA^18^. To achieve the target performance in structural applications, all these challenges must be solved to ensure that the RCA in HPC will be effective^19,20^. A viable approach to tackle this issue is the utilization of chemical and mechanical treatments of RCA prior to the addition of RCA to HPC. These are very essential in terms of increasing workability and reducing water-to-cement ratio to facilitate effective methods for concrete durability as well as strength^21^.

HPC performance is correlated to the quality and pozzolanic content of the RCA. RCA possesses high porosity, which is an undesirable characteristic that has an adverse impact on the HPC, as it reduces mechanical properties and increases water consumption. Yet, all these concerns can be overcome by appropriate chemical and mechanical treatment before using RCA in construction which can enhance workability, reduce water–cement ratio, increase the strength and durability of concrete in structural works^21^.

Various treatment methods have been proposed to enhance the performance of RCA in HPC. Chemical treatments, such as acid or sodium hydroxide (NaOH) applications, help remove weak adhered mortar and reduce porosity, thereby improving the overall quality of the aggregates^22,23^. The carbonation process, in which RCA is exposed to CO_2_, can increase its durability due to the precipitation of calcium carbonate. RCA is treated with pozzolanic slurries such as a coating of silica fume or fly ash coatings to reduce water absorption and enhance its rheological properties. Mechanical treatments such as grinder and ball milling remove adhered mortar and smoothen particle shape, although excessive grinding can negatively influence workability. SCMs like fly ash, wheat straw ash, rice husk ash, and silica fume improve the pozzolanic reaction and create a finer pore structure of RCA-based High​ Performance Concrete (HPC) giving better mechanical Properties. These materials promote interfacial transition zone densification to reduce the adverse impact of RCA and make it viable in high-performance applications^19,24^.

Using industrial solid waste contributes to reducing carbon emissions and promotes sustainability in the construction industry. Foundry sand (FS), a by-product of the metal casting industry, helps decrease the demand for natural sand, a limited resource, while minimizing industrial waste that would otherwise end up in landfills. Recycling FS to be utilized in building materials (i.e., concrete and asphalt) can minimize carbon footprints and lower potential environmental pollution. FS can also be used to improve some mechanical properties of construction materials, allowing an economical and environmentally friendly solution. Through the recycling of FS, it helps in circular economy and supports sustainability in construction^25–27^. Various researchers have explored the use of waste foundry sand (WFS) as a partial replacement of fine aggregate in concrete with conflicting results. For example, because WFS has a finer, irregular particle shape, and because it may contain unreacted binders, metals, and clay that adversely affect the hydration process of cement^28^, compressive strength may reduce significantly at higher levels of WFS contents; however, studies have shown lower replacement levels of WFS do not reduce the strength of concrete. In contrast, some researchers such as Siddique and Singh observed enhancement on the compressive strength with the increment of the WFS, especially at mid-range replacement rates^29–31^.

The use of agricultural solid waste, such as rice husk ash (RHA), in concrete has become a subject of considerable interest^32,33^. RHA, obtained from the agricultural waste rice husk after proper combustion, has about 85–95% amorphous silica^34^, which provides it with significant pozzolanic properties and high applicability in concrete. Since its particle size is very fine, RHA can effectively fill the pores in the mortar paste and interfacial transition zones (ITZs) in mortar-aggregate binding, thereby improving the overall density of concrete, and as a result, improve its properties^35,36^. Previous studies reveal that compressive strength of natural aggregate concrete (NAC) improves by substituting cement with RHA. At 28 days, the compressive strength increased by 4–16%, while at 90 days, the compressive strength increased by 4–26%^37–40^. Also improved the elastic modulus of the concrete by 14% at 28 days. Based on the literature, RHA replacement ratio generally lies within the range of 10–20%, this may also depend on the RHA material quality, especially those with high amorphous silica and fine particle size^41–45^. Rattanachu et al. and Qureshi et al.^41,46^ utilized RHA as a partial replacement material for cement (20% and 15%) in RAC where the coarse aggregates were completely substituted with RCAs. The compressive strength attained 3% and 13% higher value at 90 days, compared to the control RAC mix without inclusion of RHA.

In recent years, there have been rapid developments in artificial intelligence leading to an increased desire to implement machine learning methodologies, to predict concrete mechanical and durability properties. Compared to traditional regression approaches, these AI-based methods employ specialized algorithms that learn directly from the data, leading to more precise and reliable predictions^47–49^. For example, Peng et al.^50^ compiled a comprehensive dataset of 607 RAC samples from published literature to predict compressive strength using both traditional and hybrid machine learning models. The models included Artificial Neural Networks (ANN), Support Vector Regression (SVR), and two meta-heuristically optimized variants PSO-SVR and GWO-SVR. The highest predictive accuracy was achieved by the GWO-SVR model. Additionally, interpretability techniques such as SHAP and PDP were applied to identify key features like cement content, water content, natural fine aggregates, and water absorption, providing deeper insight into the governing factors affecting concrete performance. In the last decade, extensive research has been conducted on predicting the compressive strength of eco-friendly concrete incorporating materials such as waste glass powder, GGBS, recycled plastics in self-compacting concrete, and various industrial waste ashes. A wide range of computational techniques including artificial neural networks and nonlinear regression models have been applied with promising results^51–54^. Building upon this foundation, recent studies have advanced the application of machine learning by modeling the performance of innovative and sustainable materials, including foam glass^55^, ultra-high-performance concrete with reduced cement content^56^, geopolymer aggregates containing plastic and rubber waste^57^, and high-performance mortars incorporating rice straw ash and pumice powder^58^. However, while these studies underscore the growing potential of ML in sustainable construction, many have been oriented toward single-material systems or short-term performance evaluations. As such, there remains an opportunity to explore synergistic material interactions and long-term durability behavior under combined experimental and ML-based frameworks.

Despite substantial advances, there is still a lack of integrative research that experimentally investigates and simultaneously predicts the long-term mechanical and acid resistance performance of RAC incorporating both chemically activated agricultural waste (like RHA) and industrial by-products (such as foundry sand). Furthermore, there is limited interpretability in existing ML models, making it difficult to identify which input features most influence compressive strength outcomes. To fill these gaps, the present study develops a hybrid experimental-ML framework to evaluate and predict the compressive strength of RHA-based RAC modified with foundry sand, using SHAP and PDP for interpretability. This dual approach not only enhances understanding of material behavior but also provides a practical and explainable tool for optimizing sustainable concrete mixes over extended curing durations.

## Research novelty

In this study, a novel strategy has been introduced to enhance the performance of HPC through the incorporation of RCA (collected from 30 MPa strength concrete), chemically activated RHA and FS as partial replacements of sand. Through innovative use of industrial waste, coupled with a powerful new bio-based component, this combination not only tackles the pressing sustainability issues of the concrete industry, but also, through its use of both industrial and agricultural by-products, limits the environmental footprint of concrete manufacture itself. Unlike most published research, which mainly emphasizes the short term, the originality of this work is in the systematic long-term (up to 120 days) study of the mechanical and durability characteristics of such concrete mixtures.

In addition, the state-of-the-art machine learning and interpretable model, SHAP, and PDP methodologies are advanced in this study to understand how different material combinations affect parsing the results of permutation, which other traditional machine learning practices fail to deliver. As compressive strength is the pivotal property in HPC, this study attempts to predict the compressive strength using KNN, XGB, RF and ANN algorithms while providing insights into the material interactions that determine how concrete behaves under different conditions. Through this combination of experimental and predictive work, this dual model provides a framework for optimizing concrete formulations. The combination of extensive experimental data over multiple time frames and a long-term based machine learning prediction represents a meaningful step toward more reliable and efficient mixtures’ design for sustainable construction.

## Research methodology

**Fig. 1:**
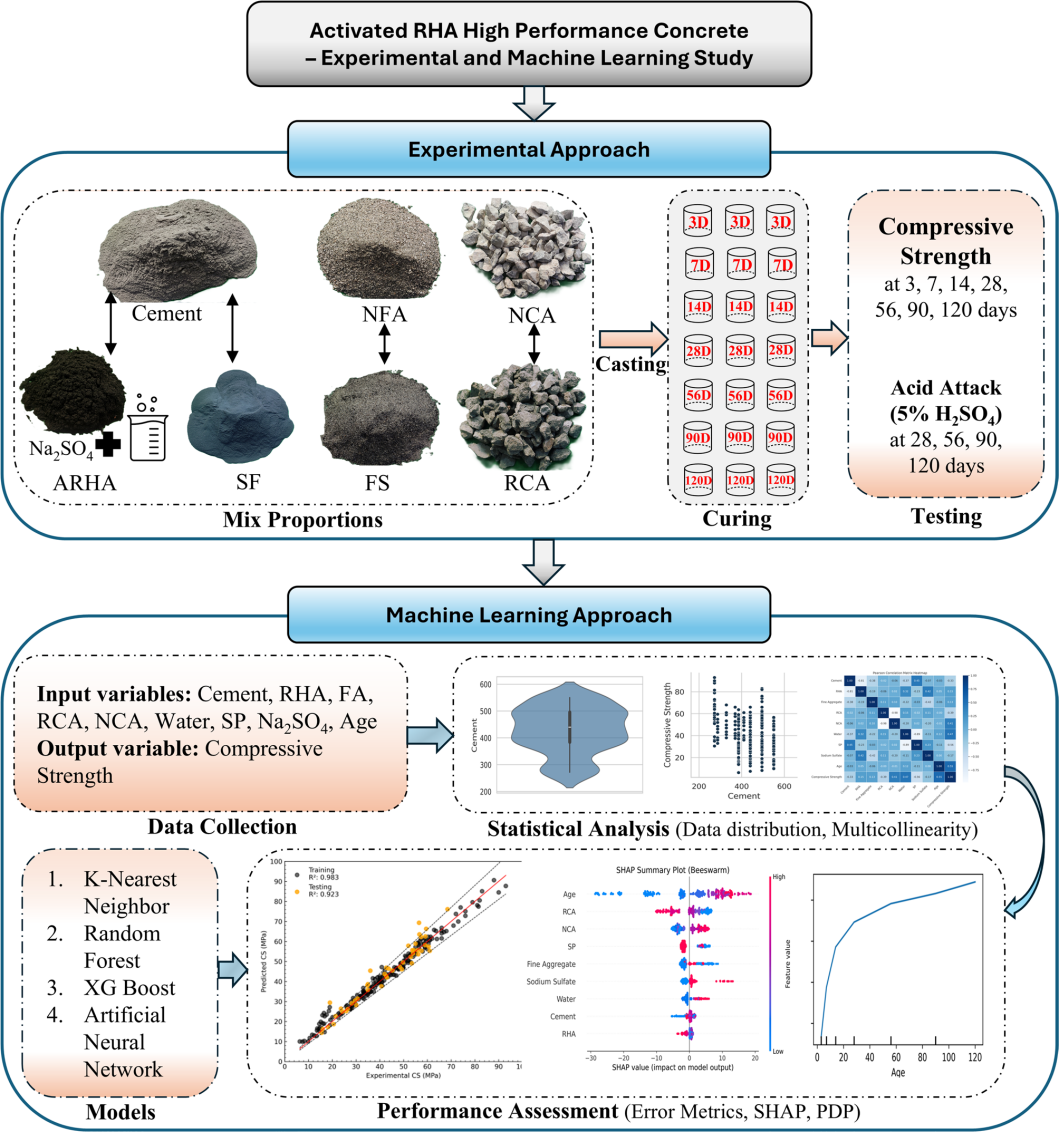
Research methodology framework.

This research integrates both experimental and machine learning approaches to evaluate the compressive strength of chemically activated RHA based RAC. The experimental phase involves material preparation, mix design, and strength testing, while the machine learning phase applies predictive modeling for performance estimation. A complete flowchart of the methodology is illustrated in Fig. 1.

### Materials

**Fig. 2:**
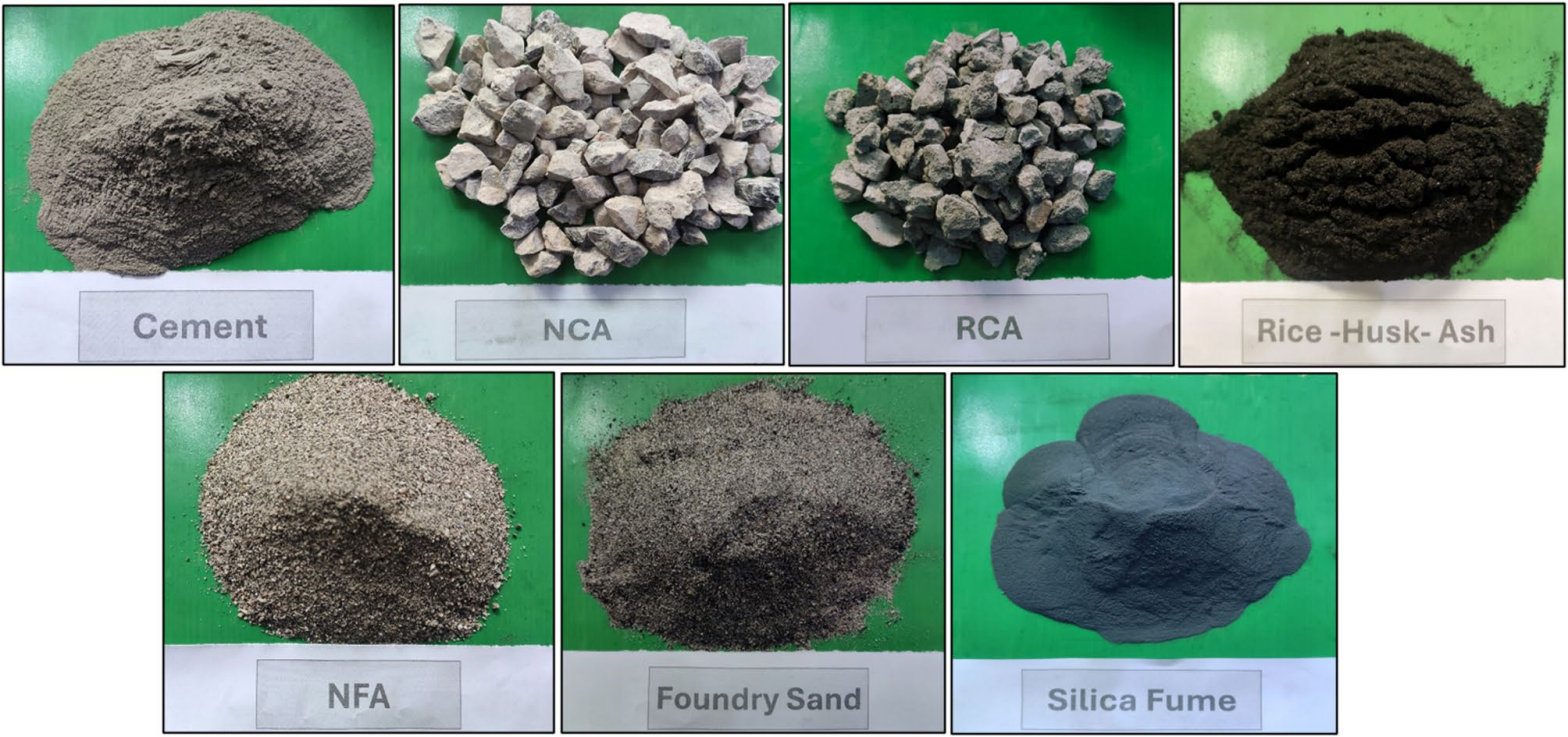
Utilization of materials in this study.

In this study, Type I OPC from the Askari brand was utilized which follows ASTM C-150 standards and is locally available in the Pakistani market. SF was collected from Imporient Chemicals, Rawalpindi, Islamabad. RHA used in this investigation was sourced from District Charsadda, KPK, Pakistan. The physical and chemical properties of the OPC are given in Table 1 and the chemical properties of the SF and RHA in Table 2. Foundry sand was sourced from local industry in Islamabad, Pakistan. The fine aggregates were procured from the Chenab River in Punjab (Pakistan) and the coarse aggregates were collected from the region of Sargodha. The physical properties of aggregates are shown in Table 3. The recycled coarse aggregates were prepared by crushing concrete cylinders with a compressive strength of 30 MPa. The crushed aggregate was passed through the sieve of 0.5 inches. The materials which are utilized in this research work are shown in Fig. 2.

**Table 1:** Properties of ordinary Portland cement.

**Chemical composition**	SiO_2_	CaO	Al_2_O_3_	SO_3_	MgO	Fe_2_O_3_	K_2_O	Na_2_O	LOI
Values (%)	20.85	64.37	4.55	1.72	1.72	3.40	0.98	0.23	2.1
**Physical properties**	Specific gravity		Blain Finess (cm^2^ /gm)		Consistency (%)		Soundness (mm)		
**Values**	3.14		3455		26.5		7		
**ASTM standards**	–		C-204		C-187		C-189		

**Table 2:** Chemical composition of silica fume and rice husk ash.

Elements	SiO_2_	CaO	Al_2_O_3_	K_2_O	Na_2_O	MgO	Fe_2_O_3_
SF	95.2%	1%	1.3%	0.15%	0.1%	0.5%	0.21%
RHA	78%	3.8%	5.7%	2.6%	1.8%	1.6%	3.84%

**Table 3:** Properties of aggregates.

Properties	Specific gravity	Absorption	Dry roded density	Loose density	Fineness modulus
**Coarse aggregate**	2.68 (ASTM C127)	0.7% (ASTM C127)	1.85 (gm/cm^3^) (ASTM C29)	1.55 (gm/cm^3^) (ASTM C29)	–
**Fine aggregate**	2.65 (ASTM C128)	2.65% (ASTM C128)	2.16 (gm/cm^3^) (ASTM C29)	2.06 (gm/cm^3^) (ASTM C29)	2.87

### Experimental work and mix proportion

The study involved the evaluation of 6 groups, each subdivided into 4 distinct subgroups with varying RHA content of 0, 10, 20 and 30%. According to the ASTM C39/C39M standard, a total of 483 concrete specimens were cast and tested for compressive strength at curing ages of 3, 7, 14, 28, 56, 90, and 120 days. For each age, the reported strength value represents the average of three tested specimens. For acid resistance evaluation in accordance with ASTM C1898, 276 specimens were immersed in 5% sulfuric acid solution and tested after 1, 2, 3 and 4 months exposure. Table 4 presented the mixed proportion of this research. The details of groups are as follows.

- Group#1: In-activated RHA.
- Group#2: Activated RHA.
- Group#3: Activated RHA + 40% RCA.
- Group#4: Activated RHA + 60% RCA.
- Group#5: Activated RHA + 80% RCA.
- Group#6: Activated RHA + 100% RCA.

All dry materials (cement, SF, RHA, aggregates, and FS) were dry mixed for 2 min using a pan mixer to ensure uniform distribution. For activated RHA mixes, Na_2_SO_4_ (3.5% by weight of binder) was first dissolved in the mixing water and then added gradually along with the superplasticizer. Wet mixing continued for another 2 min until a homogenous mix was achieved. The fresh concrete was cast into cylindrical steel molds measuring 150 mm in diameter and 300 mm in height, in two layers, and compacted using a vibrating table. After casting, the specimens were covered for 24 h, then demolded and cured in clean water at 20–25 °C until the designated testing age.

**Table 4:** Mix design of each mixture.

Mix ID	Cement	SF (Kg/m^3^)	RHA (%age)	RHA (Kg/m^3^)	FA (Kg/m^3^)	FS (Kg/m^3^)	NCA (Kg/m^3^)	RCA (Kg/m^3^)	Water (Kg/m^3^)	SP (Kg/m^3^)
RA0-RHA0	550	41.25	0	0	544	136	880	0	176	12
RA0-RHA10	495	41.25	10	55	544	136	880	0	176	12
RA0-RHA20	440	41.25	20	110	544	136	880	0	176	12
RA0-RHA30	385	41.25	30	165	544	136	880	0	176	12
RA0-RHA0-A	550	41.25	0	0	544	136	880	0	176	12
RA0-RHA10-A	495	41.25	10	55	544	136	880	0	176	12
RA0-RHA20-A	440	41.25	20	110	544	136	880	0	176	12
RA0-RHA30-A	385	41.25	30	165	544	136	880	0	176	12
RA40-RHA0-A	550	41.25	0	0	544	136	528	352	176	12
RA40-RHA10-A	495	41.25	10	55	544	136	528	352	176	12
RA40-RHA20-A	440	41.25	20	110	544	136	528	352	176	12
RA40-RHA30-A	385	41.25	30	165	544	136	528	352	176	12
RA60-RHA0-A	550	41.25	0	0	544	136	352	528	176	12
RA60-RHA10-A	495	41.25	10	55	544	136	352	528	176	12
RA60-RHA20-A	440	41.25	20	110	544	136	352	528	176	12
RA60-RHA30-A	385	41.25	30	165	544	136	352	528	176	12
RA80-RHA0-A	550	41.25	0	0	544	136	176	704	176	12
RA80-RHA10-A	495	41.25	10	55	544	136	176	704	176	12
RA80-RHA20-A	440	41.25	20	110	544	136	176	704	176	12
RA80-RHA30-A	385	41.25	30	165	544	136	176	704	176	12
RA100-RHA0-A	550	41.25	0	0	544	136	0	880	176	12
RA100-RHA10-A	495	41.25	10	55	544	136	0	880	176	12
RA100-RHA20-A	440	41.25	20	110	544	136	0	880	176	12
RA100-RHA30-A	385	41.25	30	165	544	136	0	880	176	12

### Machine learning-based modeling approach

#### Dataset characteristics and statistical analysis

This research study employed a data set comprising 233 data points, with a substantial portion sourced from the authors’ experimental work, as presented in Table 5. To enhance the variability of the dataset, supplementary data from previous studies was also incorporated^59,60^, ensuring a diverse range of input parameters. The complete dataset used in this study has been attached as a supplementary file. This step is essential because ML models can be highly sensitive to issues such as limited data variability and multicollinearity, which may compromise model stability, inflate variance, and ultimately reduce predictive accuracy. Since compressive strength is a fundamental property in evaluating the performance of concrete, this study aims to predict the compressive strength of chemically activated RHA-based recycled aggregate concrete (RAC). A total of 9 input variables were essential for the predictive modeling, which includes Cement, RHA, FA, RCA, NCA, Water, SP, Age and Na_2_SO_4_ used to activate RHA. The combination of these variables is significant as they reflect the physical, chemical, and curing properties of concrete which is key in assessing the overall performance of RHA-based RAC.

**Table 5:** Distribution of dataset points used in the study.

Ref.	Dataset points
This study	161
Fadi Althoey et al.^59^	36
Chao Liu et al.^60^	36
Total	233

**Fig. 3:**
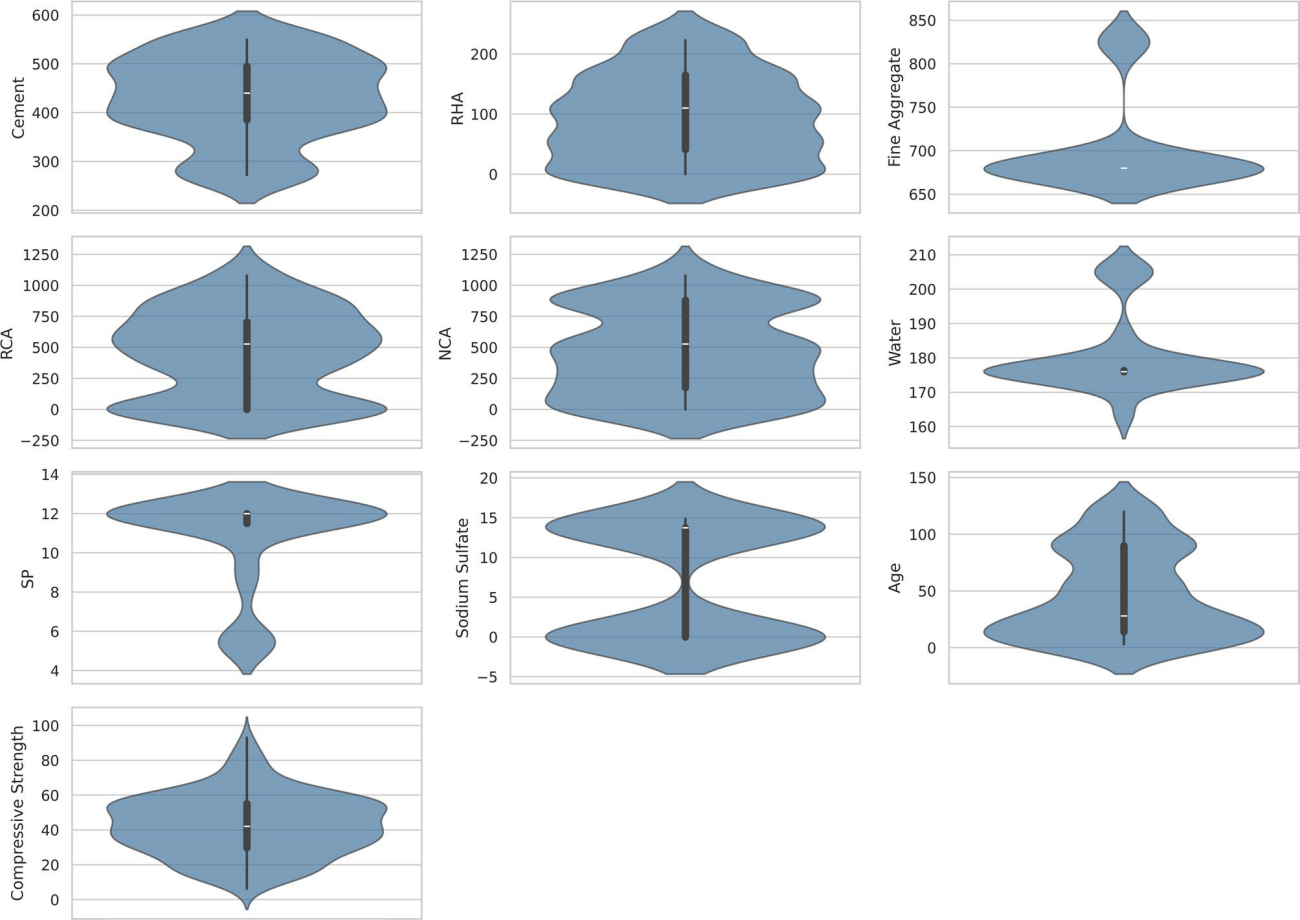
Distribution frequencies of input and output parameters.

**Fig. 4:**
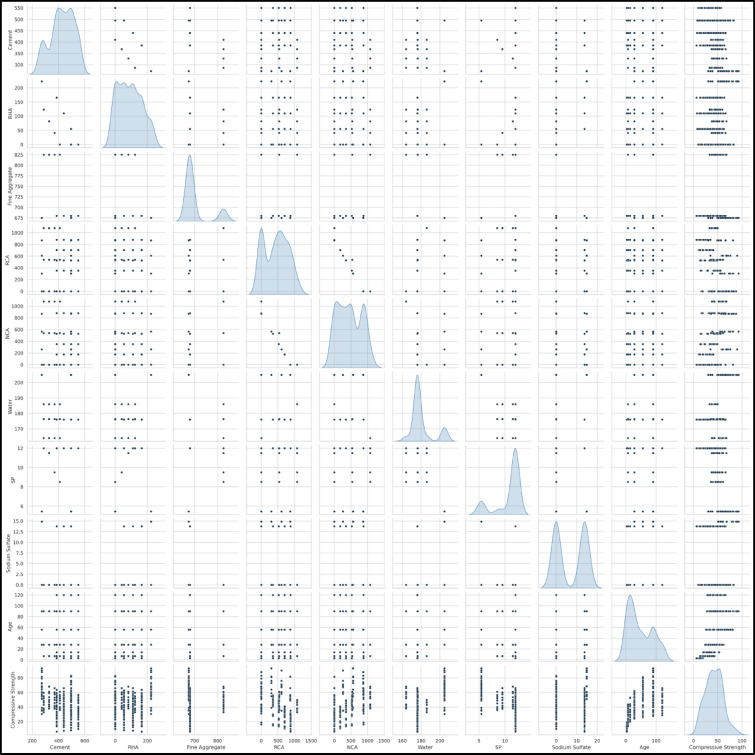
Pairwise relationships and correlations among key parameters (Cement, RHA, FA, RCA, NCA, Water, SP, Na_2_SO_4_, Age) and compressive strength.

The statistical summary for the dataset is presented in Table 6, which includes important descriptive statistics such as mean, minimum, maximum, standard deviation, variance, skewness, and kurtosis. This information could provide a view of the spread and variation of data, which an important initial step in assessing the quality of data before ML modeling. Figures 3 and 4 shows the distribution of the data as box and violin plots and pair plot respectfully, a box plot can be combined with a density plot to form a violin plot which is good for visualizing the distribution and spread of each parameter. Cement, RHA and Age have relatively symmetric distributions while RCA and NCA have broad, multi-peaked distributions, suggesting that the levels of replacement used in the mix design were diverse. The pair plot also divulges some linear (and non-linear) correlations, for instance between Age and Compressive Strength there is a positive correlation, while RCA and NCA are inversely related due to their mutual replacement.

**Table 6:** Descriptive statistics of the input variables and compressive strength.

Inputs	Mean	Minimum	Maximum	Skewness	Standard deviation	Variance	Kurtosis
Cement	427.94	272.25	550	-0.337	86.4137	7467.322	-0.845
RHA	91.93	0	222.75	0.274	72.2829	5224.821	-1.034
FA	701.63	675	825	1.919	52.8816	2796.467	1.708
RCA	448.43	0	1080	0.011	350.2647	122685.331	-1.238
NCA	460.93	0	1080	0.156	349.7863	122350.483	-1.283
Water	180.39	164	205	1.488	11.1240	123.743	1.046
SP	10.74	5.44	12	-1.593	2.4081	5.799	0.774
Sodium sulfate	6.96	0	14.85	-0.005	6.9503	48.307	-2.012
Age	46.79	3	120	0.551	39.1084	1529.469	-1.092
Compressive strength	42.68	6.32	93	0.186	17.6882	312.873	-0.277

**Fig. 5:**
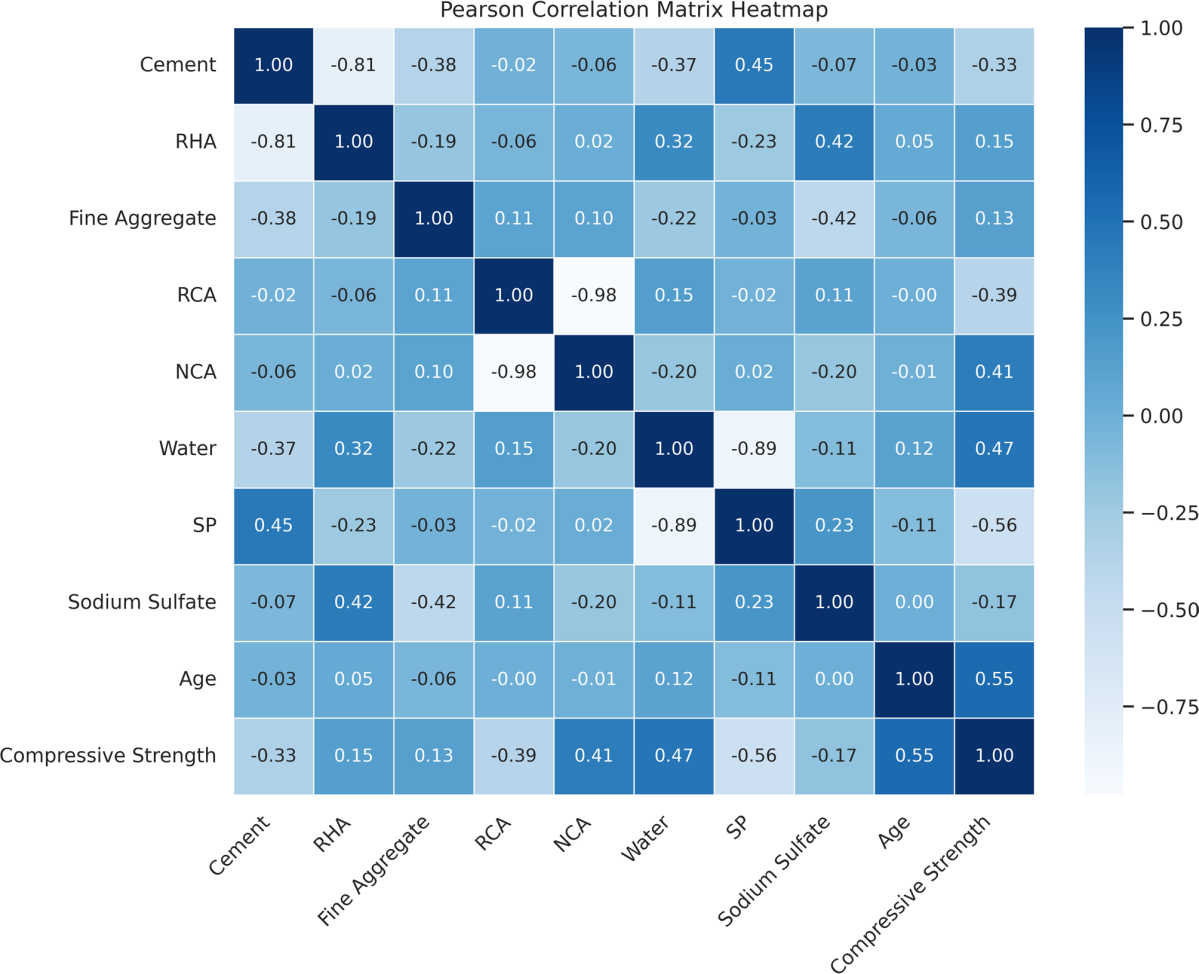
Pearson correlation matrix heatmap illustrating multicollinearity among key parameters in the concrete mix.

Furthermore, Multicollinearity, defined as a strong linear relationship between two or more independent variables, is a critical concern in regression and ML models, as it can inflate variances and distort model predictions^61,62^. Acceptable thresholds for Pearson’s correlation coefficient (R) typically lie between − 0.7 and + 0.7, with values exceeding this range indicating strong collinearity that necessitates careful examination or variable reduction^63^. The Pearson correlation heatmap in Fig. 5 highlights several notable relationships within the dataset. A high negative correlation is observed between RCA and NCA (*R* = -0.98), which is expected as RCA replaces NCA in the concrete mix. Compressive Strength demonstrates a moderate positive correlation with Age (*R* = 0.55), reflecting strength gain over time, which aligns with standard hydration processes. Additionally, Compressive Strength correlates positively with NCA (*R* = 0.41) and Water content (*R* = 0.47), while showing a moderate negative correlation with SP (*R* = -0.56), indicating the complexity of interactions between admixtures and concrete performance. A notable negative correlation is also observed between Cement and RHA (*R* = -0.81), which is anticipated due to the partial replacement nature of RHA in cementitious systems. Nevertheless, the dataset falls within the acceptable multicollinearity range. However, strong correlations between a few parameters have carefully managed through precise model optimization to ensure accurate predictions.

#### Development of predictive models and analysis

This study employed four machine learning algorithms, RF, XGB, KNN, and ANN to predict the compressive strength of chemically activated RHA-based RAC. The fundamental principles and configurations of each model are described below.

##### Random forest

The RF algorithm, developed by Breiman, is an ensemble learning technique that relies on the bagging approach, which involves bootstrapping and aggregating^64,65^. It constructs multiple decision trees during training, each on a random subset of data, and aggregates their outputs to produce the final prediction. RF is known for its robustness in handling nonlinearity, avoiding overfitting, and ensuring prediction stability through ensemble learning. Hyperparameters such as the number of trees, maximum depth, and number of features per split^66^ were fine-tuned in this study to enhance performance (Table 7).

##### Extreme gradient boosting

XGB, introduced by Chen and Guestrin, is a powerful gradient-boosting framework that builds trees sequentially, correcting residual errors from previous iterations^67–69^. It improves prediction accuracy using second-order Taylor expansion of the loss function and integrates both first and second derivatives at each step. A key advantage of XGB is its built-in regularization, which reduces overfitting and improves generalization^70,71^. In this study, XGB was used for its ability to capture complex, nonlinear interactions, and its hyperparameters such as learning rate, max depth, and boosting rounds were optimized as shown in Table 7.

##### K-nearest neighbors

The KNN algorithm is a non-parametric, instance-based method used for both regression and classification. It operates by identifying the ‘k’ closest training samples using a distance metric (e.g., Euclidean) and predicting the output as the average of those neighbors. KNN is flexible and capable of modeling local variations in data without assuming any distribution^72,73^. For this study, the optimal number of neighbors ‘k’ was determined via hyperparameter tuning (Table 7) to achieve balanced predictive accuracy.

##### Artificial neural network

ANNs are powerful models inspired by the structure and function of the human brain, capable of capturing intricate patterns in data^74^. Among various ANN types, the Feedforward Neural Network is widely used due to its straightforward yet effective architecture^75^. It comprises an input layer, one or more hidden layers, and an output layer, with information flowing in one direction from input to output through weighted connections. In this study, a Multi-Layer Perceptron was employed, using ReLU as the activation function, Adam optimizer for adaptive learning, and backpropagation for training^76–79^. The final configuration (128–64–32 neurons across three hidden layers) is provided in Table 7, enabling the model to learn nonlinear relationships for accurate compressive strength prediction.

**Table 7:** Hyperparameters of machine learning models.

Model	Key hyperparameter	Value
Random forest	Number of estimators	100
	Random state	42
XG Boost	Number of estimators	500
	Learning rate	0.03
	Max depth	4
	Min child weight	3
	Gamma	0.1
	Subsample	0.8
	Colsample by tree	0.8
	Regularization alpha (L1)	0.5
	Regularization lambda (L2)	1.0
	Random state	42
K-nearest neighbors	Number of neighbors	5
Artificial neural network	Number of hidden layers	3
	Neurons per layer	128, 64, 32
	Activation function	ReLU
	Dropout rate	0.2
	Optimizer	Adam
	Learning rate	0.001
	Loss function	MSE
	Epochs	300
	Batch size	4

##### Shapley additive explanations

SHAP additive explanation is an interpretability approach, grounded in cooperative game theory concepts^80^. It is designed to explain the predictions made by machine learning models by assigning each feature a contribution value toward the final output. SHAP values represent the marginal contribution of each feature, similar to how the Shapley value in game theory allocates payouts among players based on their individual contributions to a group effort. In the context of this study, SHAP analysis is applied to interpret the machine learning models predicting the compressive strength of RHA-based recycled aggregate concrete. This technique helps quantify the influence of each input variable, such as cement content, RHA dosage, or curing age, on the predicted compressive strength. By providing a detailed breakdown of feature importance, SHAP improves model transparency and supports more informed decision-making in optimizing concrete mix designs^80,81^.

##### Partial dependence plot

PDP visualizes the relationship between the predicted outcome of a machine learning model and one or more input features while keeping the effects of other variables averaged out. PDPs illustrate how changes in a particular feature, or a combination of features, influence the model’s prediction. This allows for a clearer understanding of the feature’s effect on the output, particularly in non-linear and complex models. In this study, PDP analysis is used to explore the influence of key variables, such as cement content, RHA, RCA replacement, and curing age, on the predicted compressive strength of concrete. By depicting the general trend between individual features and the target variable, PDP assists in unraveling complex interactions, supporting better mix proportioning, and enhancing the interpretability of the machine learning models^81^.

#### Data splitting and preprocessing

The dataset was randomly split into training and testing subsets using an 80:20 ratio, where 80% of the data was used for model training and 20% for testing. This ensures that models are trained in a sufficient portion of the data and evaluated on unseen samples to assess their generalization performance.

Data standardization was also performed to bring all input variables to a uniform scale, preventing features with larger magnitudes from dominating the learning process. The StandardScaler method from the scikit-learn library was applied, transforming the data to have a mean of zero and a standard deviation of one^82^. This preprocessing step is especially important for models like KNN and ANN, as it enhances stability and convergence during training. Standardization ultimately improves model performance and leads to more accurate and reliable predictions.

#### Evaluation parameters for model performance

The performance of machine learning models is assessed using multiple statistical metrics to ensure a comprehensive evaluation of prediction accuracy. These metrics include R² (coefficient of determination), Mean Squared Error (MSE), Root Mean Squared Error (RMSE), Normalized RMSE (NRMSE), Mean Absolute Error (MAE), and Mean Absolute Percentage Error (MAPE).

- $${R}^{2}=1-\frac{{{\Sigma}}_{i=1}^{N}{\left({x}_{i}-{\widehat{x}}_{i}\right)}^{2}}{{{\Sigma}}_{i=1}^{N}{\left({x}_{i}-\stackrel{-}{x}\right)}^{2}}$$
- $$MSE=\frac{1}{N}\sum_{i=1}^{N}{\left({x}_{i}-{\widehat{x}}_{i}\right)}^{2}$$
- $$RMSE=\sqrt{MSE}=\sqrt{\frac{1}{N}\sum_{i=1}^{N}{\left({x}_{i}-{\widehat{x}}_{i}\right)}^{2}}$$
- $$NRMSE=\frac{RMSE}{\stackrel{-}{x}}$$
- $$MAE=\frac{1}{N}\sum_{i=1}^{N}\left|{x}_{i}-{\widehat{x}}_{i}\right|$$
- $$MAPE=\frac{1}{N}\sum_{i=1}^{N}\left|\frac{{x}_{i}-{\widehat{x}}_{i}}{{x}_{i}}\right|\times100$$

## Results and discussion

### Compressive strength

**Fig. 6:**
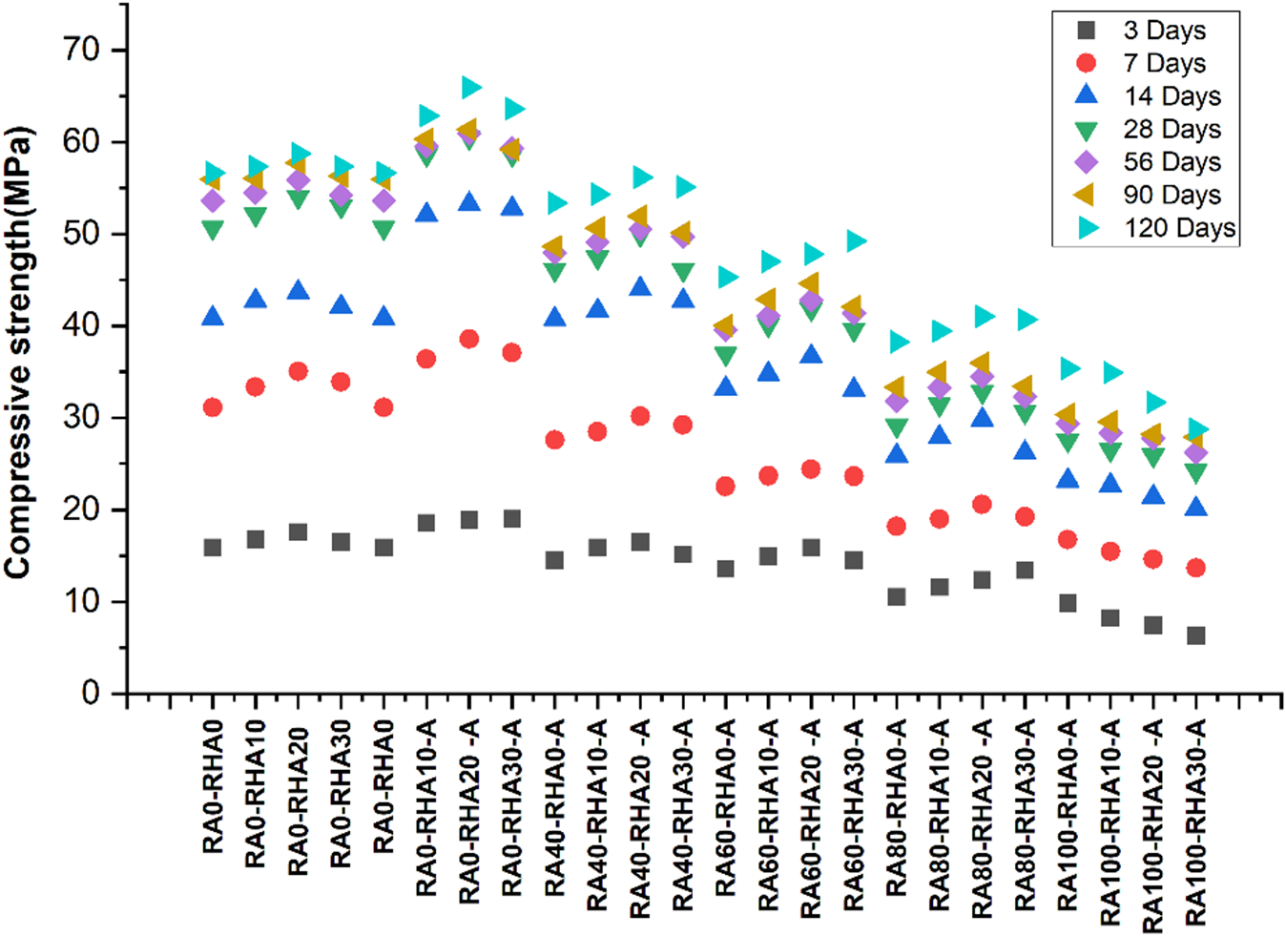
Compressive strength findings.

The findings of all mixtures are presented in Fig. 6. Group-1 is taken as a base line. For mix RA0-RHA0 at 120 days of curing time, the compressive strength increased up to 56.67 MPa gradually from 15.9 MPa at 3 days. The maximum value was observed for 20% rice husk to 58.76 MPa (RA0-RHA20) at 120 days. Similarly for Group-2 compressive strength significantly increased at all curing periods. In case of 10% RHA (RA0-RHA10-A) at 120 days of curing time, the compressive strength was found to increase up to 62.85 MPa from 18.6 MPa at 3 days. The highest increase in this group was found for 20% RHA (RA0-RHA20-A) which varied from 18.94 MPa, 38.59 MPa, 53.26 MPa,60.47 MPa, 60.95 MPa, 61.38 Mpa & 65.95 MPa with respect to the curing days (3, 7, 14, 28, 56, 90 and 120) respectively. Furthermore, in the case of 30% RHA (RA0-RHA30-A) the increase in compressive strength has been observed which varies 19.02 MPa, 37.07 MPa, 52.75 MPa, 58.62 MPa, 59.18 MPa, 59.33 Mpa & 63.64 MPa corresponding to curing days (3, 7, 14, 28, 56, 90 and 120) respectively. The reason behind chemical activation of RHA increases the reactivity, thus more CSH gel produced to fill the pore of cement paste and improve compactness. This enhanced the adhesion between the aggregates and the paste, increasing strength in early and later ages^83,84^.

Similarly in Group 3 the strength slightly decreased relative to Group 2, For mix with 0% RHA (RA40-RHA0-A) at 120 days of curing time, the compressive strength was found to increase up to 53.38 MPa from 14.52 MPa at 3 days. The highest increase in this group was also found for 20% RHA (RA40-RHA20-A) which varied from 16.54 MPa, 30.14 MPa, 44.01 MPa, 49.93 MPa, 50.52 MPa, 51.94 Mpa & 56.17 MPa with respect to curing days (3, 7, 14, 28, 56, 90 and 120) respectively. Furthermore, in the case of 30% RHA (RA40-RHA30-A) the strength has varied 15.18 MPa, 29.22 MPa, 42.7 MPa, 46.12 MPa, 49.73 MPa, 50.16 Mpa & 55.13 MPa corresponding to curing days (3, 7, 14, 28, 56, 90 and 120) respectively. These changes promoted higher porosity and weakened ITZs due to RCA incorporation, which, in turn, weakened the strength of RCA concrete. Nevertheless, the activated RHA was still effective in filling micro-pores and as a micro-filler at the ITZs despite RCA caused weaknesses^36^.

In Group 4, the strength slightly decreased relative to Group3, for mix with 0% RHA (RA60-RHA0-A) at 120 days of curing time, the strength was found to increase up to 45.29 MPa from 13.58 MPa at 3 days. The highest increase in this group was found for 20% RHA (RA60-RHA20-A) which varied from 15.91 MPa, 24.39 MPa, 36.65 MPa, 41.85 MPa, 42.81 MPa, 44.62 Mpa & 47.81 MPa with respect to curing days (3, 7, 14, 28, 56, 90 and 120) respectively. Furthermore, in the case of 30% RHA (RA60-RHA30-A) the strength varies 14.53 MPa, 23.66 MPa, 33.06 MPa, 39.58 MPa, 41.39 MPa, 42.08 Mpa & 49.24 MPa corresponding to curing days (3, 7, 14, 28, 56, 90 and 120) respectively. While the additional RCA increased porosity and degraded the ITZ, the activated RHA still contributed to promoting hydration and pozzolanic reactions over time. Despite the reduced strength, this mix demonstrates that activated RHA helps maintain long-term durability and strength to a certain extent, even with a higher RCA content. However, even when the RCA content is increased to 60%, the compressive performance of the concrete remains above 40 MPa strength at 28 days^85^.

In Group 5, the compressive strength slightly decreased relative to Group4, for mix with 0% RHA (RA80-RHA0-A) at 120 days of curing time, the compressive strength was found to increase up to 38.26 MPa gradually from 10.53 MPa at 3 days. The highest increase in this group was found to be 20% RHA (RA80-RHA20-A) which varied from 12.37 MPa, 20.59 MPa, 29.78 MPa, 32.83 MPa, 34.47 MPa, 35.94 Mpa & 41.02 MPa with respect to curing days (3, 7, 14, 28, 56, 90 and 120) respectively. Furthermore, in the case of this 30% RHA (RA80-RHA30-A) the compressive strength has been observed which varies 13.45 MPa, 19.29 MPa, 26.22 MPa, 30.65 MPa, 32.36 MPa, 33.42 Mpa & 40.67 MPa corresponding to curing days (3, 7, 14, 28, 56, 90 and 120) respectively.

Furthermore, in Group 6 the strength showed the lowest strength in comparison to all the groups, for mix with 0% RHA (RA100-RHA0-A) at 120 days of curing time, the strength was found to increase up to 35.34 MPa gradually from 9.87 MPa at 3 days. The highest compressive strength in this group was also found for 20% RHA (RA100-RHA20-A) which varied from 7.45 MPa, 14.64 MPa, 21.43 MPa, 25.89 MPa, 27.78 MPa, 28.19 Mpa & 31.67 MPa with respect to curing days (3, 7, 14, 28, 56, 90 and 120) respectively. Furthermore, in the case of this 30% RHA (RA100-RHA30-A) the strength has been varied 6.32 MPa, 13.71 MPa, 20.12 MPa, 24.23 MPa, 26.22 MPa, 27.88 MPa and 28.75 MPa corresponding to curing days (3, 7, 14, 28, 56, 90 and 120) respectively. The use of RCA for the complete replacement of natural aggregate resulted in high porosity, weaker interfacial bonds, and severe ITZ problems. The matrix density was enhanced with activated RHA; however, the porosity of the RCA limited the mix effectiveness. These results demonstrate the difficulty of employing 100% RCA for high-strength concrete, even with the use of activated RHA. However, it is still a viable solution that can be used in non-structural applications where lower strength is adequate, providing them with a sustainable solution^86^.

### Acid resistance

In this study, acid resistance was evaluated by monitoring the reduction in compressive strength of specimens exposed to sulfuric acid over time. The percentage loss in strength at each exposure period was calculated relative to the compressive strength of the corresponding 7-day water-cured specimen.

**Fig. 7:**
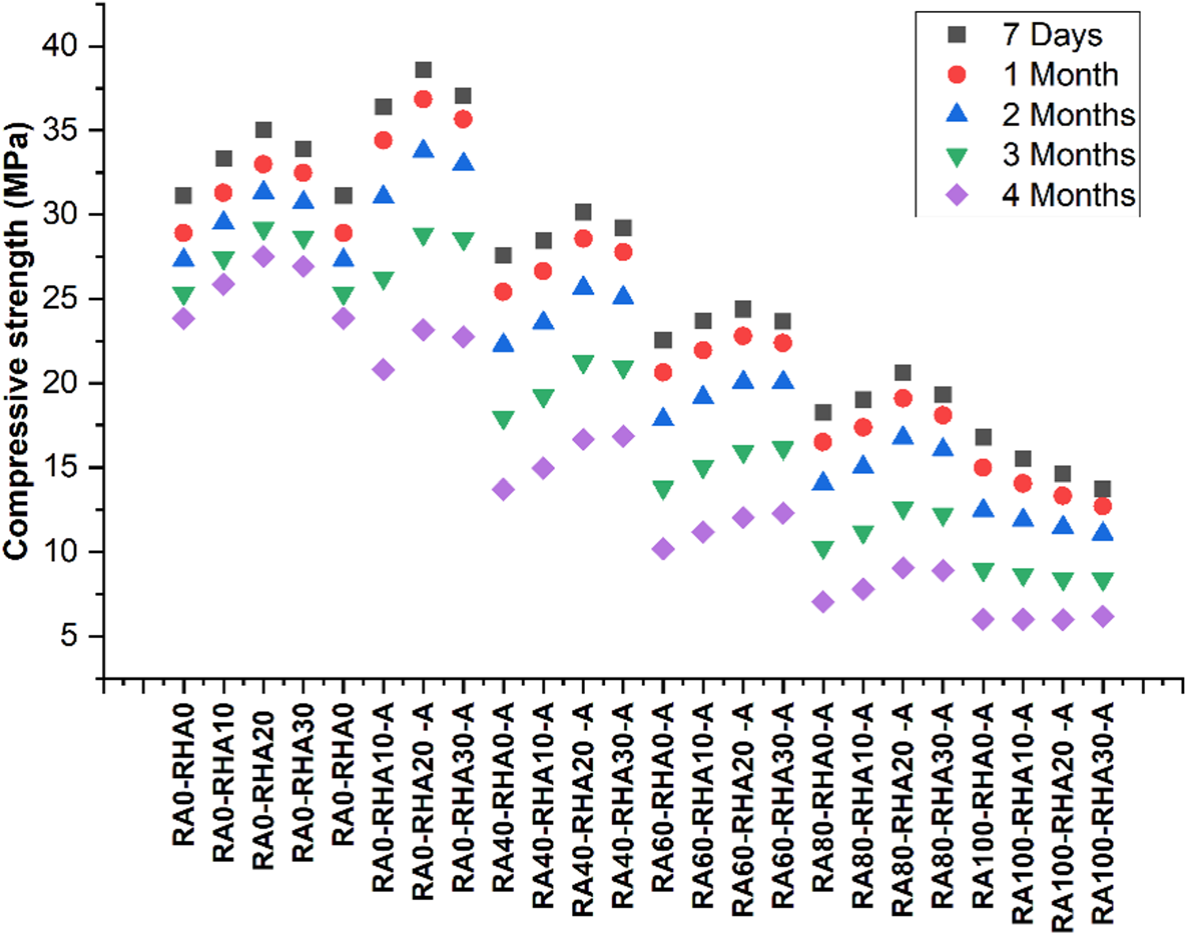
Acid resistance findings.

The acid exposure test results (Fig. 7) showed different patterns of compressive strength loss over time across six groups. In Group 1 (RA0-RHA0), the strength loss was 7.10% after 1 month, 12.32% after 2 months, 18.64% after 3 months, and 23.38% after 4 months of acid exposure. Similarly for 20% rice husk ash (RA0-RHA20) the strength loss was 5.58% at 1 month, 10.62% at 2 months, 16.74% at 3 months, and after the 4th month, strength loss had increased to 21.45%. Furthermore for 30% rice husk ash (RA0-RHA30) the Strength loss was 4.22% at 1 month, 9.41% at 2 months, and 15.45% at 3 months, while after 4th month, strength loss had increased to 20.56%.

Similarly Group-2 exhibited considerably higher resistance against acid attack. For the mix RA0-RHA10-A the Strength loss was 5.42% in 1 month, 9.78% in 2 months, 15.45% in 3 months, while after 4th month, strength loss had increased to 20.73%. In addition, 20% rice husk ash (RA0-RHA20-A) the strength loss was 4.55% at 1 month, 8.32% at 2 months, and 14.59% at 3 months, while after the 4th month, strength loss had increased to 19.67%. Furthermore for 30% rice husk ash (RA0-RHA30-A) the Strength loss was 3.83% at 1 month, 7.42% at 2 months, and 13.48% at 3 months, while after the 4th month, strength loss had increased to 20.34%. This is due to the improved pozzolanic activity of activated Rice husk ash, which leads to the densification of the concrete microstructure. So, this densification reduces acid penetration, and thereby can reduce loss of strength over time, particularly in the long term^36,84^.

Group 3, the mix having 20% rice husk ash (RA40-RHA20-A) the strength loss was 5.21% at 1 month, 10.23% at 2 months, and 17.13% at 3 months, while after the 4th month, strength loss had increased to 21.56%.Similarly for 30% rice husk ash (RA40-RHA30-A) the Strength loss was 4.92% at 1 month, 9.65% at 2 months, and 16.48% at 3 months, while after the 4th month, strength loss had increased to 19.67%.Due to the partial replacement of natural aggregates with RCA, Group 1 had a higher porosity than Group 2 specimens and therefore more vulnerable to acid penetration. Nevertheless, activated rice husk ash performed better than all inactivated mixes, indicating that some replacement of RCA is needed for rice husk ash to excel^87^.

In Group 4, containing 60% RCA, the concrete showed a lower resistance against the acid exposure. In case of 20% rice husk ash (RA60-RHA20-A) the Strength loss was 6.55% at 1 month, 11.98% at 2 months, and 20.52% at 3 months, while after the 4th month, strength loss had increased to 24.43%.Similarly for 30% rice husk ash (RA60-RHA30-A) the Strength loss was 5.46% at 1 month, 10.34% at 2 months, and 19.44% at 3 months, while after the 4th month, strength loss had increased to 23.78%. In the case of activated rice husk ash, the higher volume of RCA compromised the concrete’s microstructure and rendered it more susceptible to acid attack. This shows that although rice husk ash activation helped strengthen acid resistance, the presence of a high RCA volume lessens this protection, resulting in the acidic treatment having a greater impact on the concrete over time^87^.

Similarly Group 5 demonstrated the higher drop in strength due to a greater percentage of RCA. In the case of 20% rice husk ash (RA80-RHA20-A) the strength loss was 7.31% at 1 month, 12.15% at 2 months, and 24.67% at 3 months, while after the 4th month, strength loss had increased to 28.34%. Similarly for 30% rice husk ash (RA80-RHA30-A) the Strength loss was 6.17% at 1 month, 11.34% at 2 months, and 23.75% at 3 months, while after the 4th month, strength loss had increased to 27.22%. In Group 6, containing 100% RCA, the maximum strength drop was observed. In the case of 20% rice husk ash (RA100-RHA20-A) the Strength loss was 8.83% at 1 month, 14.11% at 2 months, and 26.63% at 3 months, while after the 4th month, strength loss had increased to 28.84%.Similarly for 30% rice husk ash (RA100-RHA30-A) the Strength loss was 7.19% at 1 month, 12.82% at 2 months, and 24.31% at 3 months, while after the 4th month, strength loss had increased to 26.22%.The extensive replacement of natural aggregates with RCA resulted in a very porous matrix, which allowed increased acid ingress and consequently, more significant strength loss, even considering the presence of activated RHA. The result indicates that activated RHA can enhance durability; however, its role fades dramatically with higher amounts of RCA, particularly in complete RCA substitution^88^.

### ML models’ assessment and performance

#### Performance metrics

**Fig. 8:**
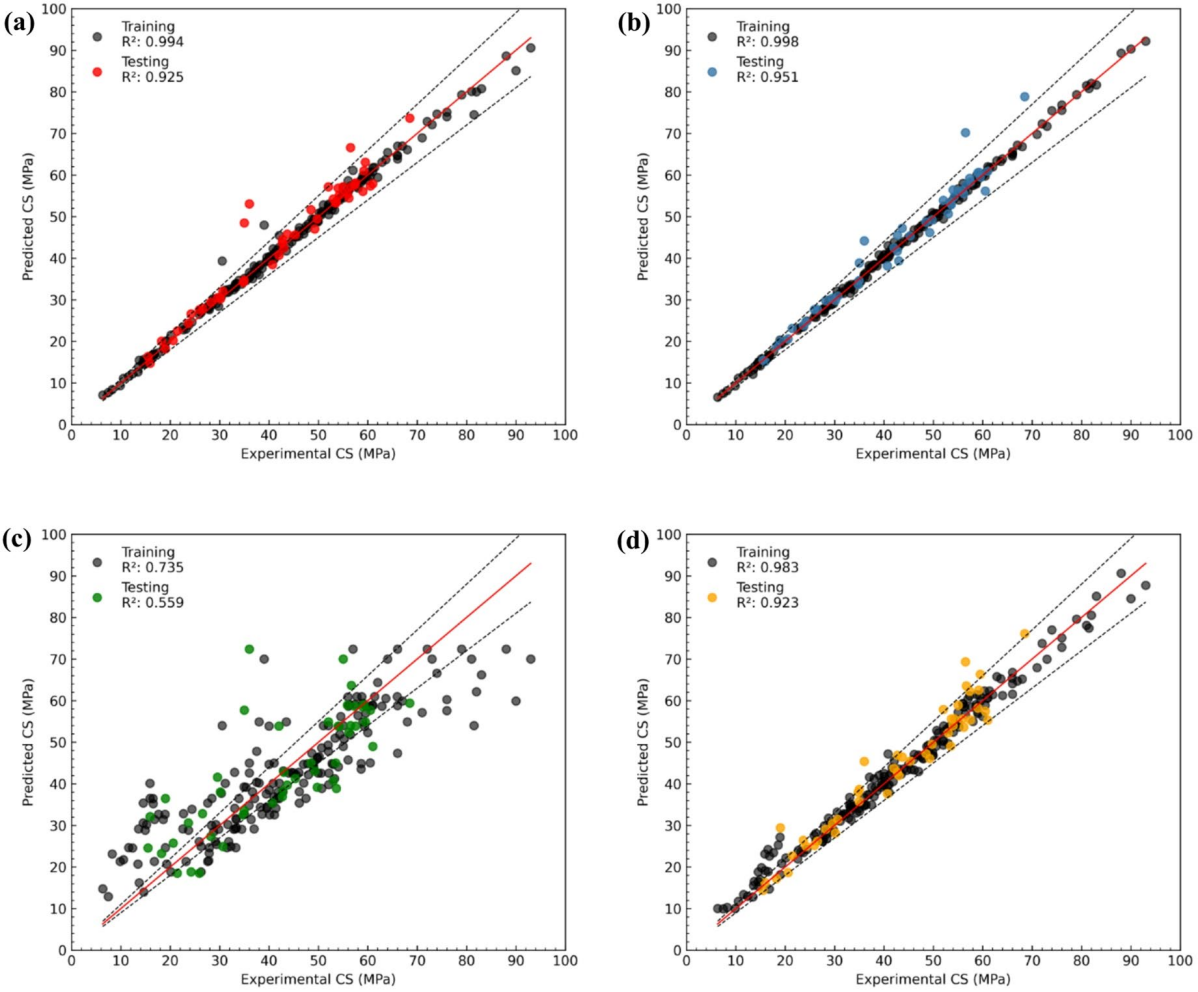
Regression plots comparing KNN, RF, XGB, and ANN models in predicting compressive strength (CS), showing experimental vs. predicted values with R² for training and testing datasets. (**a**) RF, (**b**) XGB, (**c**) KNN, (**d**) ANN.

The predictive accuracy of four machine learning models RF, XGB, KNN, and ANN evaluated to estimate the compressive strength of chemically activated RHA-based RAC. The performance of these models was assessed based on R^2^ values for both training and testing datasets, as shown in Fig. 8a–d. These figures compare the predicted compressive strength (y-axis) with the actual experimental values (x-axis), where the solid black line represents a perfect prediction line (R^2^ = 1), and dashed lines denote a ± 10% error margin.

The RF model (Fig. 8a) demonstrated strong predictive capability, achieving an R^2^ value of 0.994 for training and 0.925 for testing. Most data points clustered closely along the perfect prediction line, with predictions well within the ± 10% margin, suggesting the robustness of the RF model. The XGB model (Fig. 8b) exhibited the highest accuracy among the models, with R^2^ values of 0.998 for training and 0.951 for testing sets. Predictions were densely grouped around the ideal line, with negligible deviation, reflecting XGB’s superior generalization ability and precision. The KNN model (Fig. 8c) exhibited the lowest performance, with R^2^ values of 0.735 (training) and 0.559 (testing). Predictions were widely scattered, particularly at higher compressive strengths, indicating weaker reliability and a tendency to underfit complex patterns in the dataset as compared to XGB and RF. These findings are well aligned with the literature^70,89^. Lastly, the ANN model (Fig. 8d) produced R^2^ values of 0.983 for training and 0.923 for testing, signifying strong predictive power, though slightly trailing behind RF and XGB. Despite being a powerful model, ANN can sometimes encounter issues such as overfitting, where it memorizes training data patterns excessively, or underfitting, where it struggles to generalize complex relationships. Additionally, ANN’s performance is highly sensitive to hyperparameter selection, network architecture, and data quantity, which can lead to slight deviations compared to tree-based models like XGB.

Additionally, Table 8 summarizes the performance metrics R^2^, MSE, RMSE, NRMSE, MAE, and MAPE for all models during training and testing. The XGB model consistently recorded the lowest errors, with an RMSE of 0.718 (train) and 3.222 (test), and an MAPE of 1.489% (train) and 4.299% (test). RF followed closely with slightly higher error margins, while ANN performed competitively, particularly in testing. Conversely, the KNN model yielded the highest RMSE (9.686) and MAPE (21.192%) during testing, highlighting its limitations in this application. This underperformance of KNN can be attributed to its sensitivity to local data density and noise, which poses challenges in complex, high-dimensional regression problems like concrete strength prediction.

**Table 8:** Performance evaluation of random Forest, XG Boost, KNN, and ANN models during the training and testing phases.

	Evaluation parameters	Random forest	XG Boost	KNN	ANN
Training phase (80%)	R^2^	0.994	0.998	0.735	0.983
	MSE	2.132	0.515	89.28	5.654
	RMSE	1.460	0.718	9.449	2.378
	NRMSE	0.017	0.008	0.109	0.027
	MAE	0.840	0.54	7.201	1.824
	MAPE %	2.211	1.489	24.55	6.142
Testing phase (20%)	R^2^	0.925	0.951	0.559	0.923
	MSE	15.87	10.38	93.81	16.46
	RMSE	3.984	3.222	9.686	4.058
	NRMSE	0.075	0.061	0.183	0.077
	MAE	2.310	1.862	7.046	2.872
	MAPE %	5.815	4.299	21.19	7.184

#### Taylor diagram

**Fig. 9:**
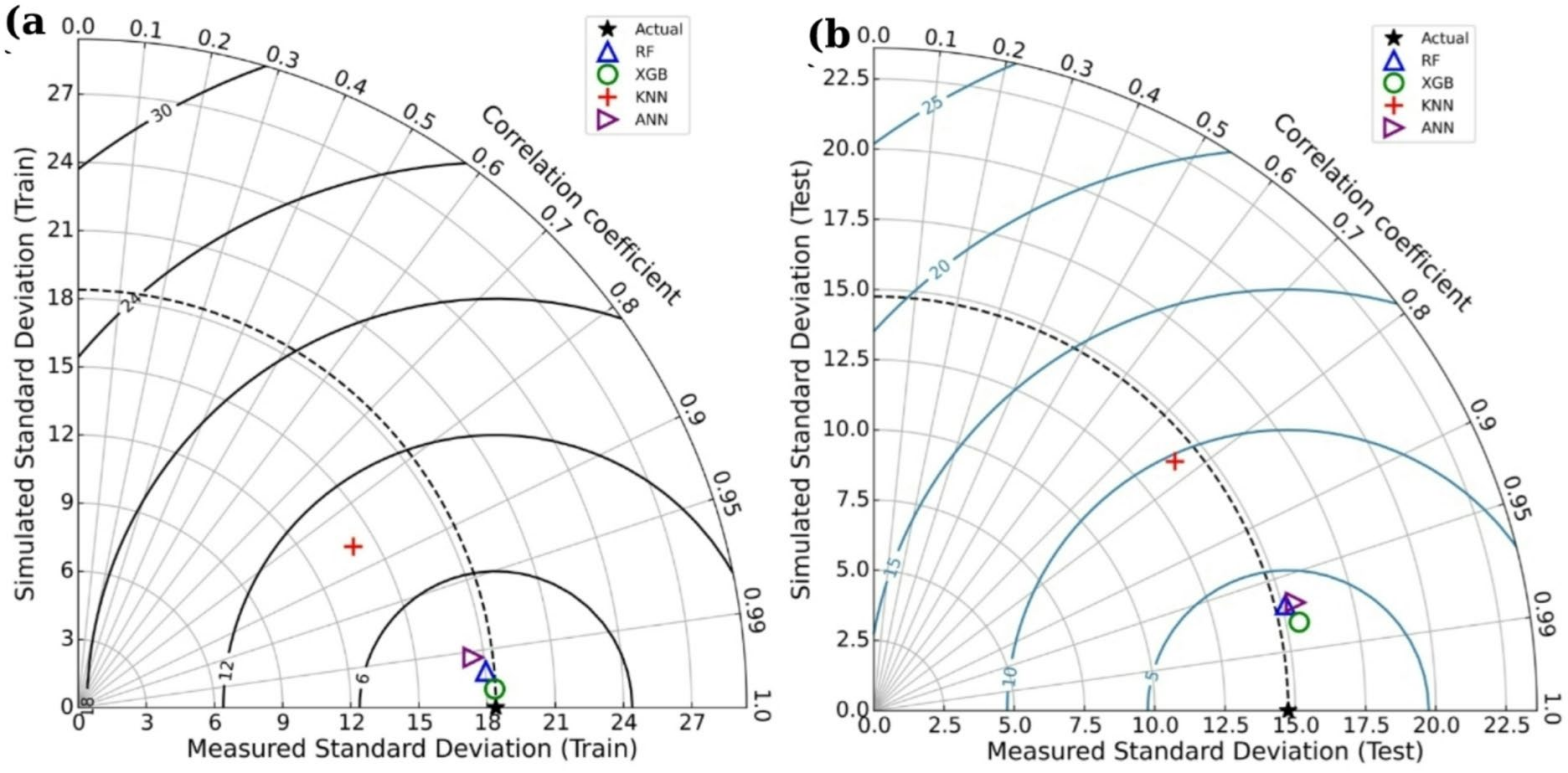
Taylor diagrams for (**a**) training and (**b**) testing phases illustrating the correlation, standard deviation, and RMSE of predictions.

Taylor diagrams (Fig. 9a, b) offer a graphical comparison of model performance by assessing the correlation coefficient (R), standard deviation, and centered RMSE. The diagram for the training phase (Fig. 9a) indicates that XGB closely matched the reference point (black star), with a correlation coefficient near 0.998 and a standard deviation closely aligned with the experimental data. RF and ANN also performed well, clustering near XGB, while KNN displayed a lower correlation coefficient and greater deviation.

For the testing phase (Fig. 9b), the XGB model maintained its superior performance, with a correlation coefficient of approximately 0.95. RF and ANN models followed closely, while KNN remained the least accurate, with a lower correlation and a more substantial deviation from the reference. The combined evaluation of scatter plots, performance metrics, and Taylor diagrams highlights the XGB model’s superior predictive capability, followed by RF and ANN, while KNN demonstrated limited accuracy, particularly for high compressive strength values.

#### SHAP analysis

**Fig. 10:**
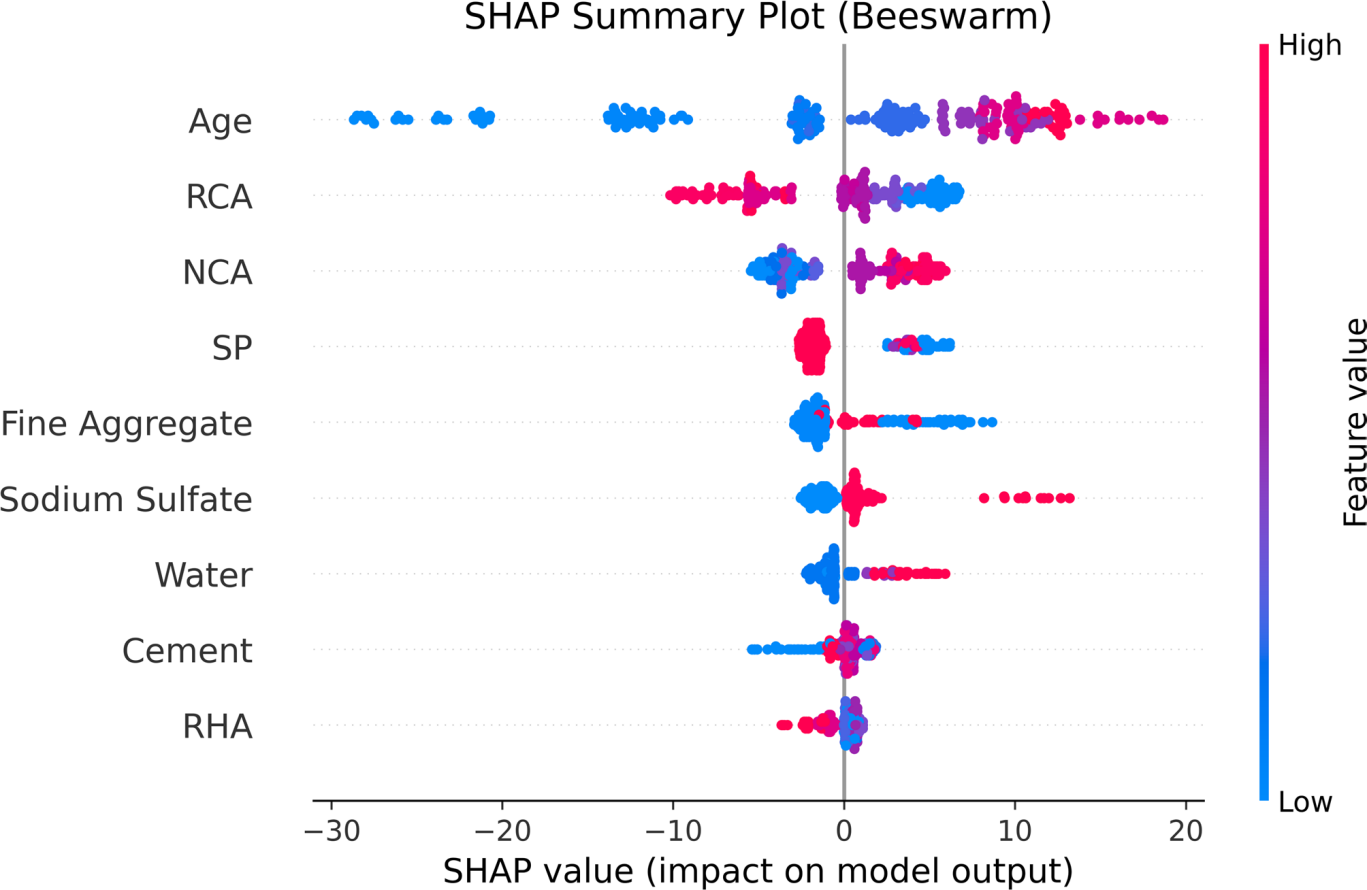
SHAP summary plot illustrating the impact of each feature on model predictions, ranked by importance.

**Fig. 11:**
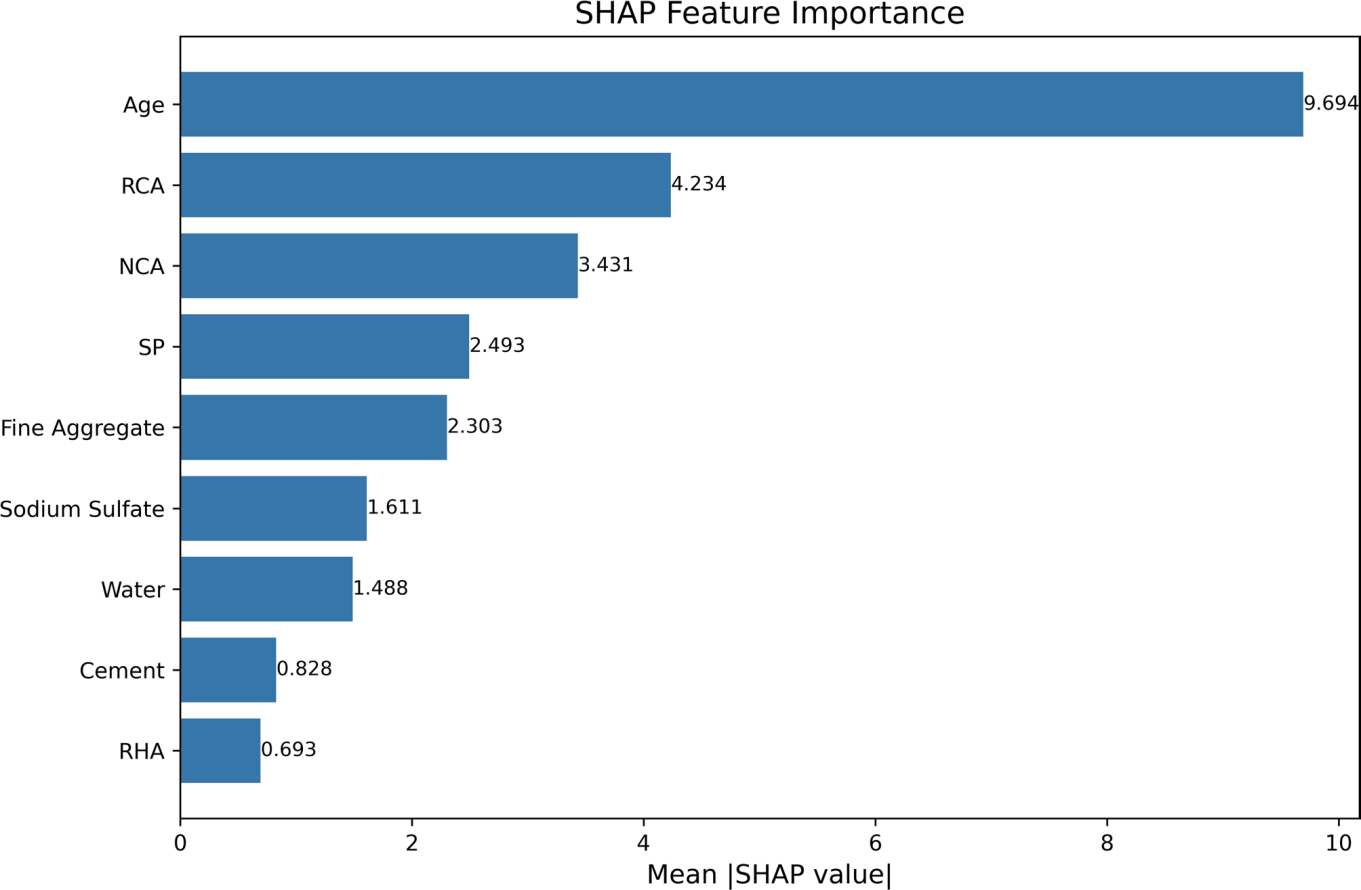
Feature importance derived from SHAP values, highlighting the most influential variables in the model.

SHAP analysis was employed to evaluate the influence of each input feature on the prediction of compressive strength for chemically activated RHA-based RAC. Figure 10 presents the SHAP summary plot, while Fig. 11 illustrates the mean absolute SHAP values in a bar plot, indicating the overall importance of each parameter.

The SHAP summary and bar plots highlight curing age as the most influential factor with a SHAP value ≈ 9.7, which aligns with the established understanding that extended curing improves hydration and results in greater strength development. RCA and NCA follow with SHAP values of 4.234 and 3.431 respectively, underscoring the importance of aggregate quality and type in concrete performance. Notably, the SHAP summary plot for RCA reveals a high frequency of negative SHAP values, suggesting a predominantly decreasing effect on compressive strength. This observation is consistent with the experimental results, where RCA generally exhibited a lowering effect on strength compared to natural aggregates.

SP and FA also exhibit substantial influence, with SHAP values of 2.493 and 2.303, respectively. These contributions reflect their role in enhancing workability and optimizing particle packing, which improve both the fresh and hardened properties of concrete. Na_2_SO_4_ with SHAP value of 1.61, representing the chemical activation of RHA, shows a positive impact on compressive strength, validating its role as an activator in enhancing the pozzolanic reaction and improving the binding properties of the matrix. Cement content (0.828) and water content (1.488) exhibit relatively lower importance, likely due to the balanced mix design approach where other materials, such as SP and RHA, manage workability and strength development. Among these, RHA appears with the lowest mean SHAP value (0.693), yet it still demonstrates a detectable influence on CS prediction. In this study, RHA was incorporated as a partial replacement for cement, where an increase in RHA proportion corresponded to a decrease in cement content. This inverse relationship may have contributed to the overall lower importance score in the SHAP bar plot. However, the SHAP summary plot (Fig. 10) reveals that RHA has a positive impact on strength up to a certain threshold, beyond which the effect shifts toward neutral or even negative. This observation aligns with known behavior of pozzolanic materials, where optimal replacement levels enhance matrix densification and strength, while excessive substitution can dilute the binder phase.

Furthermore, the interaction between RHA and curing age likely plays a critical role in long-term performance. The pozzolanic reaction of RHA is time-dependent, often contributing more significantly to strength development at later curing stages. This synergy explains why curing age exhibited the highest SHAP value (9.694), reinforcing that RHA’s strength-enhancing potential is amplified with extended curing. Therefore, even with a relatively lower standalone SHAP score, RHA remains a key supplementary component whose effectiveness is closely tied to the curing regime.

#### Partial dependence plots

**Fig. 12:**
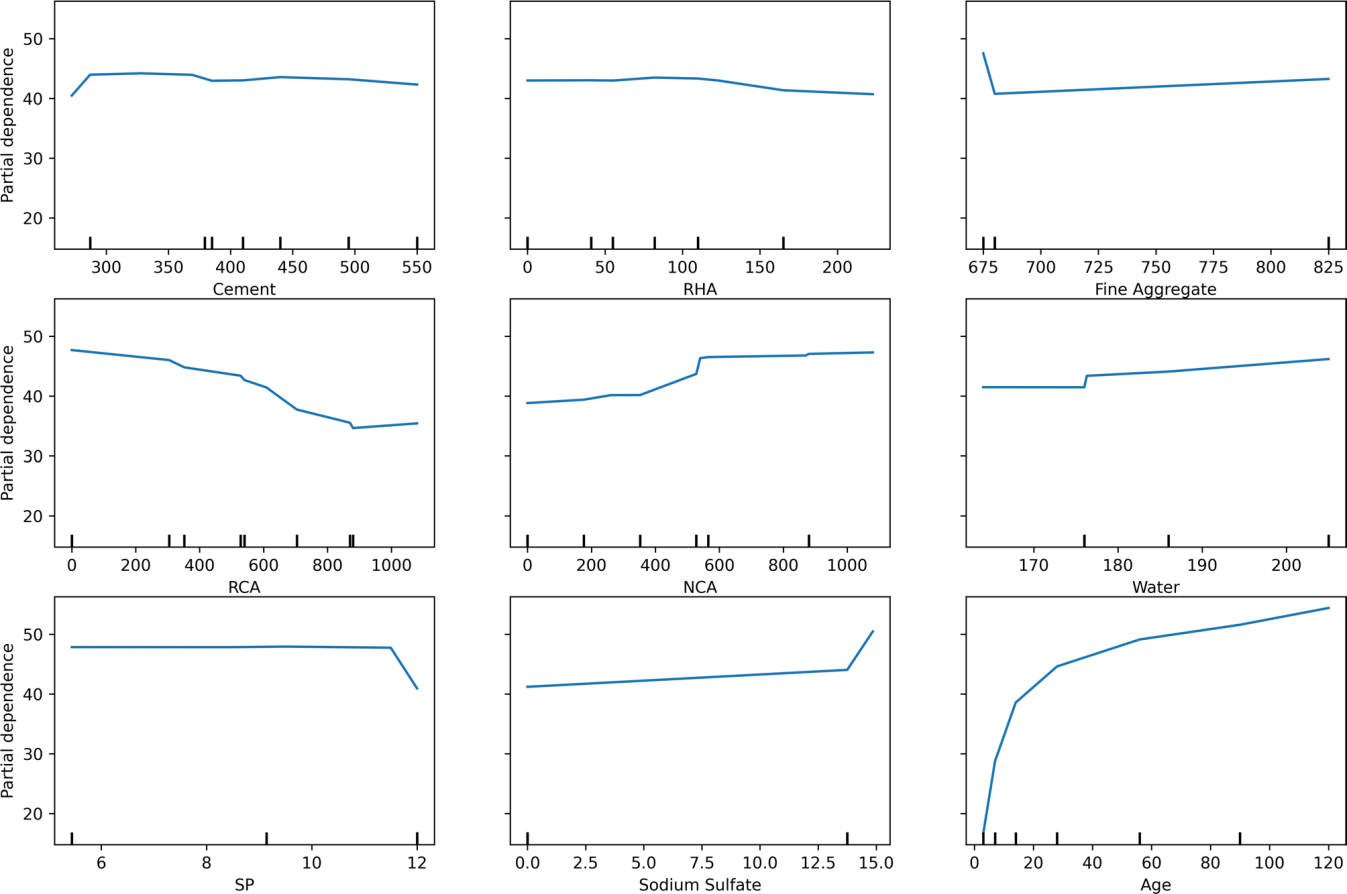
Partial Dependence Plots showing the relationship between key features and predicted outcomes.

PDPs were generated to investigate the marginal influence of each input variable on the predicted compressive strength of chemically activated RHA-based RAC, as depicted in Fig. 12. These plots provide an intuitive visualization of how individual changes in specific variables affect the model’s output, while keeping all other inputs constant, offering valuable insights into the behavior of the predictive model.

The cement content plot reveals an initial positive contribution to compressive strength as dosage increases; however, a plateau is observed beyond approximately 450–500 kg/m^3^, indicating diminishing returns. This suggests that excessive cement content may not significantly enhance strength beyond a certain threshold. RHA exhibits a relatively flat response, indicating a minimal direct impact on strength within the tested range. This is understandable, as RHA replacements were relatively constant across the dataset; however, its underlying pozzolanic contribution is still detected within the system, as shown in the SHAP analysis. Fine Aggregate content displays a subtle positive trend, suggesting its effect is relatively neutral within the tested range, provided the mix maintains proper workability and particle gradation.

The RCA plot reveals a notable negative impact on strength, particularly beyond 400 kg/m^3^, aligning with experimental findings. This behavior can be attributed to RCA’s weaker ITZ and higher porosity, which compromise the overall concrete matrix strength. Conversely, NCA demonstrates a positive influence on strength, stabilizing after approximately 600 kg/m^3^, which reflects its structural contribution to enhancing the load-bearing capacity. Water content exhibits a mild upward trend, likely supported by the presence of SP ensuring workability, thereby avoiding the typical strength reduction associated with excess water. SP itself shows an overall neutral effect, indicating its primary role as a workability agent within the tested range, rather than directly affecting strength development. Na_2_SO_4_, acting as an activator for RHA, exhibits a positive impact on strength, particularly at higher dosages, validating its role in enhancing the pozzolanic reactivity and overall cementitious properties. Curing age emerges as the most influential variable, displaying a steep increase in strength up to 40 days, followed by a gradual stabilization, reflecting the sustained hydration process and pozzolanic reactions of RHA over time.

The PDP analysis strongly aligns with experimental observations and SHAP interpretations, highlighting curing age, RCA, and NCA as the most critical parameters influencing compressive strength in chemically activated RHA-based RAC. This consistency across multiple analytical approaches reinforces the reliability of the developed machine learning models and underscores the need to carefully optimize these key variables when designing sustainable RAC mixtures incorporating RHA. Furthermore, the model uncovers nuanced thresholds and interactions such as the diminishing effect of cement beyond 500 kg/m^3^ and the nonlinear degradation due to RCA that may not be easily identified through traditional empirical testing. The neutral behavior of superplasticizer and the detected positive impact of Na_2_SO_4_ activation offer additional clarity on dosage optimization. These findings not only align with known material behavior but also provide quantifiable, interpretable patterns that help validate and fine-tune sustainable concrete mix designs. Thus, the model serves as a complementary tool for both validating domain knowledge and revealing actionable data-driven insights.

#### Comparative evaluation with previous studies

To assess the robustness and relevance of the developed models, a comparative analysis was conducted against recent studies that employed machine learning for compressive strength prediction of sustainable and recycled concretes, as summarized in Table 9. One such study focused on foam glass, a lightweight material known for its insulation and impact-resistant properties^55^. Using a dataset of 214 samples, the authors applied Gradient Boosting, Random Forest, Gaussian Process Regression (GPR), and Linear Regression to predict porosity and compressive strength. Among these, the GPR model achieved the best performance (*R* = 0.91 for porosity and 0.82 for compressive strength), with density and foam agent content identified as the most influential features through partial dependence analysis. Peng et al.^50^ developed a large database of 607 RAC samples and applied both traditional (ANN, SVR) and hybrid models (PSO-SVR, GWO-SVR). Their results showed that GWO-SVR attained the highest accuracy (R^2^ = 0.9056), while feature importance techniques such as SHAP and PDP identified cement content, water content, and aggregate properties as influential.

In a study on UHPC by^56^, XGB was applied to mixes containing silica fume and steel fibers, resulting in an R^2^ of 0.901 and RMSE of 11.52 MPa. The study effectively leveraged feature importance methods to support mix optimization. Compared to these contributions, the present study achieved a high-test accuracy (R^2^ = 0.951, RMSE = 3.22 MPa) using XGB, supported by KNN, RF and ANN models. The dataset was derived from a combination of experimental work and literature, encompassing a unique material system of RCA, chemically activated RHA, and foundry sand. Additionally, the inclusion of long-term curing data (up to 120 days) and the use of SHAP and PDP analyses for interpretability offer added value. Together, these aspects contribute to an adaptable and explainable modeling framework that aligns with current sustainability objectives in the concrete industry.

**Table 9:** Comparative summary of ML-based studies for compressive strength prediction in sustainable concrete systems.

Author/Year	Dataset details	ML models used	Target variable(s)	Best model & performance	Key insights
Peng et al. [50] / 2023	*N* = 607; RAC; mix proportion, material properties	ANN, SVR, PSO-SVR, GWO-SVR	CS	GWO-SVR – R^2^ = 0.9056 (overall), but overfitting noted	Hybrid models outperformed basic ML; SHAP & PDP identified cement, water, and NFA as key inputs
Abdellatief et al. [55]/ 2018	*N* = 214; foam glass; inputs: glass particle diameter, foam agent content, heating rate, dry density, etc.	GB, RF, GPR, LR	Porosity, CS	GPR – *R* = 0.91 (porosity), 0.82 (CS)	GPR outperformed others; density and foam agent content were most influential
Abdellatief et al. [56] / 2025	*N* = 357; UHPC; inputs: GGBS, SF, limestone powder, fly ash, QP, nano silica etc.	XGB, RF, GB, GPR	CS	XGB – R^2^ ≈ 0.901, RMSE ≈ 11.52 MPa	Highlighted SF and fiber content; ML used to optimize eco-friendly UHPC mix
This study	*N* = 233; RHA-based RAC with FS and RCA (up to 120 days)	XGB, RF, ANN, KNN	CS	XGB – R^2^ = 0.951 (test), RMSE = 3.22 MPa	Superior accuracy: SHAP and PDP used for model interpretability; long-term dataset

## Future recommendations

Future studies are focused on determining the best percentage of RCA and examining different chemicals to improve the strength of RHA concrete. Investigating innovative treatment methods for RCA (such as carbonation and mechanical grinding methods) can lead to increased aggregate quality and concrete durability. Long-term performance tests under different environmental conditions such as freeze-thaw and sulfate exposure tests are also needed to validate its performance for structural applications. The dataset advancement for machine-learning models and application of more sophisticated algorithm, e.g. deep learning, may enhance the prediction accuracy and offer insightful understanding of concrete performance. However, a full life cycle assessment must be conducted to quantify the environmentally beneficial aspects of incorporating chemically activated RHA and RCA into the construction industry.

## Conclusion

This study investigated the compressive strength of chemically activated RHA-based RAC through both experimental testing and ML approaches. The main findings are summarized below:

- The compressive strength of concrete increased consistently with curing time across all mixtures, confirming the effectiveness of long-term hydration and pozzolanic activity of RHA.
- In mixes without chemical activation, a 20% RHA replacement led to the highest strength of 58.76 MPa at 120 days, compared to 56.67 MPa for the control mix without RHA.
- Chemical activation of RHA significantly improved compressive strength. At 20% RHA, the strength rose from 18.94 MPa (3 days) to 65.95 MPa (120 days), a substantial enhancement over inactivated mixes.
- The inclusion of recycled aggregates led to a progressive decline in strength; however, activated RHA effectively mitigated this reduction.
- At 60% RCA replacement, the best-performing mix achieved 41.85 MPa at 28 days and 47.81 MPa at 120 days, which remains suitable for structural applications.
- Even with 100% RCA, the mix with 20% activated RHA achieved 25.89 MPa at 28 days and improved to 31.67 MPa at 120 days, showing promise for non-structural or low-strength applications.
- All mixes experienced progressive strength loss over the 4-month acid exposure period, with the rate of degradation increasing with time and recycled aggregate content.
- Chemically activated RHA significantly improved acid resistance; at 4 months, the strength loss reduced from 23.38% (control) to 19.67% for the 20% activated RHA mix, demonstrating enhanced durability due to improved matrix densification.
- Even with partial RCA inclusion (up to 40%), activated RHA helped maintain strength loss below 22%, highlighting its effectiveness in moderately recycled systems.
- However, at 100% RCA, acid resistance declined sharply, with strength loss reaching 28.84%, indicating that the protective role of activated RHA diminishes with excessive RCA due to increased porosity and compromised microstructure.
- Overall, the optimal performance was achieved with 20% chemically activated RHA, which significantly enhanced compressive strength (reaching 65.95 MPa at 120 days) and improved acid resistance. Even with up to 40% RCA, the strength loss after 4 months of acid exposure remained comparable to the control mix without RHA activation, indicating that activated RHA effectively enhances both mechanical performance and durability even in the presence of recycled aggregates.
- Among the four ML models evaluated, XGB demonstrated the highest prediction accuracy with an R^2^ of 0.951, RMSE of 3.222 MPa, and MAE of 1.862 MPa.
- SHAP analysis identified curing age as the most influential variable (mean SHAP ≈ 10), followed by RCA (3.54), NCA (3.27), and superplasticizer (SP) (1.91).
- Despite a relatively low SHAP value (0.693), RHA still showed a measurable influence, particularly due to its interaction with activators like Na_2_SO_4_.
- Partial dependence plots confirmed the nonlinear and synergistic influence of multiple variables, especially RCA content and curing age, on compressive strength.

This study contributes to the growing body of knowledge on hybrid ML-experimental frameworks for predicting and enhancing the strength of green concrete. The proposed methodology can serve as a foundation for future work involving other supplementary cementitious materials and long-term durability performance.

## Supplementary Information

Below is the link to the electronic supplementary material.

Supplementary Material 1

## Data Availability

The data will be available upon request to corresponding author.

## Abbreviations

HPC: High performance concrete
RCA: Recycled coarse aggregates
RAC: Recycled aggregate concrete
NAC: Natural aggregate concrete
RA: Recycled aggregates
FA: Fine aggregate concrete
RHA: Rice husk ash
FS: Foundry sand
WFS: Waste foundry sand
SF: Silica fume
KNN: K nearest neighbor
RF: Random forest
XGB: Extreme gradient boosting
ANN: Artificial neural network
SHAP: SHapley additive explanations
PDP: Partial dependence plots
C&D: Construction and demolition
SCMs: Supplementary cementitious materials
ML: Machine learning
OPC: Ordinary portland cement
SP: Superplasticizer
MSE: Mean squared error
RMSE: Root mean squared error
NRMSE: Normalized root mean squared error
MAE: Mean absolute error
MAPE: Mean absolute percentage error
