# Supplementary material for: Experimental and machine learning prediction of compressive strength of chemically activated RHA based RAC using SHAP and PDP analysis
Source: Sci Rep. 2025 Dec 4;15:43164. doi: 10.1038/s41598-025-25592-2 (PMC12678409; doi:10.1038/s41598-025-25592-2)
Supplement: Supplementary file 1 — Supplementary Material 1 [file 41598_2025_25592_MOESM1_ESM.docx]

**Experimental and Machine Learning Prediction of Compressive Strength of Chemically Activated RHA based RAC using SHAP and PDP Analysis**

**Supplementary File**

1. **Data Source References with Access Links**

This provides a detailed list of the original dataset sources used in this study. Each reference includes a direct link to publicly available data for easy access.

1. Current Study
2. Fadi Althoey et. al. [59]. Available at: <https://doi.org/10.1016/j.jobe.2022.104610>
3. Chao Liu et. al. [60]. Available at: <https://doi.org/10.1016/j.conbuildmat.2022.128934>

**S2. Dataset:**

| **Sr. No.** | **Cement**  **(kg/m^3^)** | **RHA**  **(kg/m^3^)** | **FA**  **(kg/m^3^)** | **RCA**  **(kg/m^3^)** | **NCA**  **(kg/m^3^)** | **Water**  **(kg/m^3^)** | **SP**  **(kg/m^3^)** | **Sodium Sulfate**  **(kg/m^3^)** | **Age**  **(days)** | **CS**  **(MPa)** |
| --- | --- | --- | --- | --- | --- | --- | --- | --- | --- | --- |
|  | 550 | 0 | 680 | 0 | 880 | 176 | 12 | 0 | 3 | **15.9** |
|  | 495 | 55 | 680 | 0 | 880 | 176 | 12 | 0 | 3 | **16.8** |
|  | 440 | 110 | 680 | 0 | 880 | 176 | 12 | 0 | 3 | **17.62** |
|  | 385 | 165 | 680 | 0 | 880 | 176 | 12 | 0 | 3 | **16.52** |
|  | 550 | 0 | 680 | 0 | 880 | 176 | 12 | 0 | 7 | **31.12** |
|  | 495 | 55 | 680 | 0 | 880 | 176 | 12 | 0 | 7 | **33.34** |
|  | 440 | 110 | 680 | 0 | 880 | 176 | 12 | 0 | 7 | **35.03** |
|  | 385 | 165 | 680 | 0 | 880 | 176 | 12 | 0 | 7 | **33.9** |
|  | 550 | 0 | 680 | 0 | 880 | 176 | 12 | 0 | 14 | **40.82** |
|  | 495 | 55 | 680 | 0 | 880 | 176 | 12 | 0 | 14 | **42.73** |
|  | 440 | 110 | 680 | 0 | 880 | 176 | 12 | 0 | 14 | **43.68** |
|  | 385 | 165 | 680 | 0 | 880 | 176 | 12 | 0 | 14 | **42.06** |
|  | 550 | 0 | 680 | 0 | 880 | 176 | 12 | 0 | 28 | **50.66** |
|  | 495 | 55 | 680 | 0 | 880 | 176 | 12 | 0 | 28 | **52.11** |
|  | 440 | 110 | 680 | 0 | 880 | 176 | 12 | 0 | 28 | **54.04** |
|  | 385 | 165 | 680 | 0 | 880 | 176 | 12 | 0 | 28 | **53.01** |
|  | 550 | 0 | 680 | 0 | 880 | 176 | 12 | 0 | 56 | **53.64** |
|  | 495 | 55 | 680 | 0 | 880 | 176 | 12 | 0 | 56 | **54.51** |
|  | 440 | 110 | 680 | 0 | 880 | 176 | 12 | 0 | 56 | **55.86** |
|  | 385 | 165 | 680 | 0 | 880 | 176 | 12 | 0 | 56 | **54.22** |
|  | 550 | 0 | 680 | 0 | 880 | 176 | 12 | 0 | 90 | **55.97** |
|  | 495 | 55 | 680 | 0 | 880 | 176 | 12 | 0 | 90 | **56.08** |
|  | 440 | 110 | 680 | 0 | 880 | 176 | 12 | 0 | 90 | **57.77** |
|  | 385 | 165 | 680 | 0 | 880 | 176 | 12 | 0 | 90 | **56.33** |
|  | 550 | 0 | 680 | 0 | 880 | 176 | 12 | 0 | 120 | **56.67** |
|  | 495 | 55 | 680 | 0 | 880 | 176 | 12 | 0 | 120 | **57.34** |
|  | 440 | 110 | 680 | 0 | 880 | 176 | 12 | 0 | 120 | **58.76** |
|  | 385 | 165 | 680 | 0 | 880 | 176 | 12 | 0 | 120 | **57.35** |
|  | 495 | 55 | 680 | 0 | 880 | 176 | 12 | 13.75 | 3 | **18.6** |
|  | 440 | 110 | 680 | 0 | 880 | 176 | 12 | 13.75 | 3 | **18.94** |
|  | 385 | 165 | 680 | 0 | 880 | 176 | 12 | 13.75 | 3 | **19.02** |
|  | 495 | 55 | 680 | 0 | 880 | 176 | 12 | 13.75 | 7 | **36.39** |
|  | 440 | 110 | 680 | 0 | 880 | 176 | 12 | 13.75 | 7 | **38.59** |
|  | 385 | 165 | 680 | 0 | 880 | 176 | 12 | 13.75 | 7 | **37.07** |
|  | 495 | 55 | 680 | 0 | 880 | 176 | 12 | 13.75 | 14 | **52.07** |
|  | 440 | 110 | 680 | 0 | 880 | 176 | 12 | 13.75 | 14 | **53.26** |
|  | 385 | 165 | 680 | 0 | 880 | 176 | 12 | 13.75 | 14 | **52.75** |
|  | 495 | 55 | 680 | 0 | 880 | 176 | 12 | 13.75 | 28 | **58.65** |
|  | 440 | 110 | 680 | 0 | 880 | 176 | 12 | 13.75 | 28 | **60.47** |
|  | 385 | 165 | 680 | 0 | 880 | 176 | 12 | 13.75 | 28 | **58.62** |
|  | 495 | 55 | 680 | 0 | 880 | 176 | 12 | 13.75 | 56 | **59.55** |
|  | 440 | 110 | 680 | 0 | 880 | 176 | 12 | 13.75 | 56 | **60.95** |
|  | 385 | 165 | 680 | 0 | 880 | 176 | 12 | 13.75 | 56 | **59.33** |
|  | 495 | 55 | 680 | 0 | 880 | 176 | 12 | 13.75 | 90 | **60.33** |
|  | 440 | 110 | 680 | 0 | 880 | 176 | 12 | 13.75 | 90 | **61.38** |
|  | 385 | 165 | 680 | 0 | 880 | 176 | 12 | 13.75 | 90 | **59.18** |
|  | 495 | 55 | 680 | 0 | 880 | 176 | 12 | 13.75 | 120 | **62.85** |
|  | 440 | 110 | 680 | 0 | 880 | 176 | 12 | 13.75 | 120 | **65.95** |
|  | 385 | 165 | 680 | 0 | 880 | 176 | 12 | 13.75 | 120 | **63.64** |
|  | 550 | 0 | 680 | 352 | 528 | 176 | 12 | 0 | 3 | **14.52** |
|  | 495 | 55 | 680 | 352 | 528 | 176 | 12 | 13.75 | 3 | **15.93** |
|  | 440 | 110 | 680 | 352 | 528 | 176 | 12 | 13.75 | 3 | **16.54** |
|  | 385 | 165 | 680 | 352 | 528 | 176 | 12 | 13.75 | 3 | **15.18** |
|  | 550 | 0 | 680 | 352 | 528 | 176 | 12 | 0 | 7 | **27.56** |
|  | 495 | 55 | 680 | 352 | 528 | 176 | 12 | 13.75 | 7 | **28.45** |
|  | 440 | 110 | 680 | 352 | 528 | 176 | 12 | 13.75 | 7 | **30.14** |
|  | 385 | 165 | 680 | 352 | 528 | 176 | 12 | 13.75 | 7 | **29.22** |
|  | 550 | 0 | 680 | 352 | 528 | 176 | 12 | 0 | 14 | **40.73** |
|  | 495 | 55 | 680 | 352 | 528 | 176 | 12 | 13.75 | 14 | **41.63** |
|  | 440 | 110 | 680 | 352 | 528 | 176 | 12 | 13.75 | 14 | **44.01** |
|  | 385 | 165 | 680 | 352 | 528 | 176 | 12 | 13.75 | 14 | **42.7** |
|  | 550 | 0 | 680 | 352 | 528 | 176 | 12 | 0 | 28 | **46.12** |
|  | 495 | 55 | 680 | 352 | 528 | 176 | 12 | 13.75 | 28 | **47.46** |
|  | 440 | 110 | 680 | 352 | 528 | 176 | 12 | 13.75 | 28 | **49.93** |
|  | 385 | 165 | 680 | 352 | 528 | 176 | 12 | 13.75 | 28 | **46.12** |
|  | 550 | 0 | 680 | 352 | 528 | 176 | 12 | 0 | 56 | **47.97** |
|  | 495 | 55 | 680 | 352 | 528 | 176 | 12 | 13.75 | 56 | **49.12** |
|  | 440 | 110 | 680 | 352 | 528 | 176 | 12 | 13.75 | 56 | **50.52** |
|  | 385 | 165 | 680 | 352 | 528 | 176 | 12 | 13.75 | 56 | **49.73** |
|  | 550 | 0 | 680 | 352 | 528 | 176 | 12 | 0 | 90 | **48.64** |
|  | 495 | 55 | 680 | 352 | 528 | 176 | 12 | 13.75 | 90 | **50.64** |
|  | 440 | 110 | 680 | 352 | 528 | 176 | 12 | 13.75 | 90 | **51.94** |
|  | 385 | 165 | 680 | 352 | 528 | 176 | 12 | 13.75 | 90 | **50.16** |
|  | 550 | 0 | 680 | 352 | 528 | 176 | 12 | 0 | 120 | **53.38** |
|  | 495 | 55 | 680 | 352 | 528 | 176 | 12 | 13.75 | 120 | **54.33** |
|  | 440 | 110 | 680 | 352 | 528 | 176 | 12 | 13.75 | 120 | **56.17** |
|  | 385 | 165 | 680 | 352 | 528 | 176 | 12 | 13.75 | 120 | **55.13** |
|  | 550 | 0 | 680 | 528 | 352 | 176 | 12 | 0 | 3 | **13.58** |
|  | 495 | 55 | 680 | 528 | 352 | 176 | 12 | 13.75 | 3 | **14.94** |
|  | 440 | 110 | 680 | 528 | 352 | 176 | 12 | 13.75 | 3 | **15.91** |
|  | 385 | 165 | 680 | 528 | 352 | 176 | 12 | 13.75 | 3 | **14.53** |
|  | 550 | 0 | 680 | 528 | 352 | 176 | 12 | 0 | 7 | **22.54** |
|  | 495 | 55 | 680 | 528 | 352 | 176 | 12 | 13.75 | 7 | **23.70** |
|  | 440 | 110 | 680 | 528 | 352 | 176 | 12 | 13.75 | 7 | **24.39** |
|  | 385 | 165 | 680 | 528 | 352 | 176 | 12 | 13.75 | 7 | **23.66** |
|  | 550 | 0 | 680 | 528 | 352 | 176 | 12 | 0 | 14 | **33.17** |
|  | 495 | 55 | 680 | 528 | 352 | 176 | 12 | 13.75 | 14 | **34.76** |
|  | 440 | 110 | 680 | 528 | 352 | 176 | 12 | 13.75 | 14 | **36.65** |
|  | 385 | 165 | 680 | 528 | 352 | 176 | 12 | 13.75 | 14 | **33.06** |
|  | 550 | 0 | 680 | 528 | 352 | 176 | 12 | 0 | 28 | **36.95** |
|  | 495 | 55 | 680 | 528 | 352 | 176 | 12 | 13.75 | 28 | **40.11** |
|  | 440 | 110 | 680 | 528 | 352 | 176 | 12 | 13.75 | 28 | **41.85** |
|  | 385 | 165 | 680 | 528 | 352 | 176 | 12 | 13.75 | 28 | **39.58** |
|  | 550 | 0 | 680 | 528 | 352 | 176 | 12 | 0 | 56 | **39.56** |
|  | 495 | 55 | 680 | 528 | 352 | 176 | 12 | 13.75 | 56 | **41.09** |
|  | 440 | 110 | 680 | 528 | 352 | 176 | 12 | 13.75 | 56 | **42.81** |
|  | 385 | 165 | 680 | 528 | 352 | 176 | 12 | 13.75 | 56 | **41.39** |
|  | 550 | 0 | 680 | 528 | 352 | 176 | 12 | 0 | 90 | **40.02** |
|  | 495 | 55 | 680 | 528 | 352 | 176 | 12 | 13.75 | 90 | **42.88** |
|  | 440 | 110 | 680 | 528 | 352 | 176 | 12 | 13.75 | 90 | **44.62** |
|  | 385 | 165 | 680 | 528 | 352 | 176 | 12 | 13.75 | 90 | **42.08** |
|  | 550 | 0 | 680 | 528 | 352 | 176 | 12 | 0 | 120 | **45.29** |
|  | 495 | 55 | 680 | 528 | 352 | 176 | 12 | 13.75 | 120 | **46.99** |
|  | 440 | 110 | 680 | 528 | 352 | 176 | 12 | 13.75 | 120 | **47.81** |
|  | 385 | 165 | 680 | 528 | 352 | 176 | 12 | 13.75 | 120 | **49.24** |
|  | 550 | 0 | 680 | 704 | 176 | 176 | 12 | 0 | 3 | **10.53** |
|  | 495 | 55 | 680 | 704 | 176 | 176 | 12 | 13.75 | 3 | **11.58** |
|  | 440 | 110 | 680 | 704 | 176 | 176 | 12 | 13.75 | 3 | **12.37** |
|  | 385 | 165 | 680 | 704 | 176 | 176 | 12 | 13.75 | 3 | **13.45** |
|  | 550 | 0 | 680 | 704 | 176 | 176 | 12 | 0 | 7 | **18.24** |
|  | 495 | 55 | 680 | 704 | 176 | 176 | 12 | 13.75 | 7 | **19.02** |
|  | 440 | 110 | 680 | 704 | 176 | 176 | 12 | 13.75 | 7 | **20.59** |
|  | 385 | 165 | 680 | 704 | 176 | 176 | 12 | 13.75 | 7 | **19.29** |
|  | 550 | 0 | 680 | 704 | 176 | 176 | 12 | 0 | 14 | **25.86** |
|  | 495 | 55 | 680 | 704 | 176 | 176 | 12 | 13.75 | 14 | **27.91** |
|  | 440 | 110 | 680 | 704 | 176 | 176 | 12 | 13.75 | 14 | **29.78** |
|  | 385 | 165 | 680 | 704 | 176 | 176 | 12 | 13.75 | 14 | **26.22** |
|  | 550 | 0 | 680 | 704 | 176 | 176 | 12 | 0 | 28 | **29.12** |
|  | 495 | 55 | 680 | 704 | 176 | 176 | 12 | 13.75 | 28 | **31.45** |
|  | 440 | 110 | 680 | 704 | 176 | 176 | 12 | 13.75 | 28 | **32.83** |
|  | 385 | 165 | 680 | 704 | 176 | 176 | 12 | 13.75 | 28 | **30.65** |
|  | 550 | 0 | 680 | 704 | 176 | 176 | 12 | 0 | 56 | **31.81** |
|  | 495 | 55 | 680 | 704 | 176 | 176 | 12 | 13.75 | 56 | **33.28** |
|  | 440 | 110 | 680 | 704 | 176 | 176 | 12 | 13.75 | 56 | **34.47** |
|  | 385 | 165 | 680 | 704 | 176 | 176 | 12 | 13.75 | 56 | **32.26** |
|  | 550 | 0 | 680 | 704 | 176 | 176 | 12 | 0 | 90 | **33.32** |
|  | 495 | 55 | 680 | 704 | 176 | 176 | 12 | 13.75 | 90 | **34.96** |
|  | 440 | 110 | 680 | 704 | 176 | 176 | 12 | 13.75 | 90 | **35.94** |
|  | 385 | 165 | 680 | 704 | 176 | 176 | 12 | 13.75 | 90 | **33.42** |
|  | 550 | 0 | 680 | 704 | 176 | 176 | 12 | 0 | 120 | **38.26** |
|  | 495 | 55 | 680 | 704 | 176 | 176 | 12 | 13.75 | 120 | **39.43** |
|  | 440 | 110 | 680 | 704 | 176 | 176 | 12 | 13.75 | 120 | **41.02** |
|  | 385 | 165 | 680 | 704 | 176 | 176 | 12 | 13.75 | 120 | **40.67** |
|  | 550 | 0 | 680 | 880 | 0 | 176 | 12 | 0 | 3 | **9.87** |
|  | 495 | 55 | 680 | 880 | 0 | 176 | 12 | 13.75 | 3 | **8.23** |
|  | 440 | 110 | 680 | 880 | 0 | 176 | 12 | 13.75 | 3 | **7.45** |
|  | 385 | 165 | 680 | 880 | 0 | 176 | 12 | 13.75 | 3 | **6.32** |
|  | 550 | 0 | 680 | 880 | 0 | 176 | 12 | 0 | 7 | **16.78** |
|  | 495 | 55 | 680 | 880 | 0 | 176 | 12 | 13.75 | 7 | **15.49** |
|  | 440 | 110 | 680 | 880 | 0 | 176 | 12 | 13.75 | 7 | **14.64** |
|  | 385 | 165 | 680 | 880 | 0 | 176 | 12 | 13.75 | 7 | **13.71** |
|  | 550 | 0 | 680 | 880 | 0 | 176 | 12 | 0 | 14 | **23.18** |
|  | 495 | 55 | 680 | 880 | 0 | 176 | 12 | 13.75 | 14 | **22.68** |
|  | 440 | 110 | 680 | 880 | 0 | 176 | 12 | 13.75 | 14 | **21.43** |
|  | 385 | 165 | 680 | 880 | 0 | 176 | 12 | 13.75 | 14 | **20.12** |
|  | 550 | 0 | 680 | 880 | 0 | 176 | 12 | 0 | 28 | **27.56** |
|  | 495 | 55 | 680 | 880 | 0 | 176 | 12 | 13.75 | 28 | **26.51** |
|  | 440 | 110 | 680 | 880 | 0 | 176 | 12 | 13.75 | 28 | **25.89** |
|  | 385 | 165 | 680 | 880 | 0 | 176 | 12 | 13.75 | 28 | **24.23** |
|  | 550 | 0 | 680 | 880 | 0 | 176 | 12 | 0 | 56 | **29.36** |
|  | 495 | 55 | 680 | 880 | 0 | 176 | 12 | 13.75 | 56 | **28.33** |
|  | 440 | 110 | 680 | 880 | 0 | 176 | 12 | 13.75 | 56 | **27.78** |
|  | 385 | 165 | 680 | 880 | 0 | 176 | 12 | 13.75 | 56 | **26.22** |
|  | 550 | 0 | 680 | 880 | 0 | 176 | 12 | 0 | 90 | **30.34** |
|  | 495 | 55 | 680 | 880 | 0 | 176 | 12 | 13.75 | 90 | **29.56** |
|  | 440 | 110 | 680 | 880 | 0 | 176 | 12 | 13.75 | 90 | **28.19** |
|  | 385 | 165 | 680 | 880 | 0 | 176 | 12 | 13.75 | 90 | **27.88** |
|  | 550 | 0 | 680 | 880 | 0 | 176 | 12 | 0 | 120 | **35.34** |
|  | 495 | 55 | 680 | 880 | 0 | 176 | 12 | 13.75 | 120 | **34.91** |
|  | 440 | 110 | 680 | 880 | 0 | 176 | 12 | 13.75 | 120 | **31.67** |
|  | 385 | 165 | 680 | 880 | 0 | 176 | 12 | 13.75 | 120 | **28.75** |
|  | 495 | 0 | 675 | 0 | 870 | 205 | 5.44 | 0 | 28 | **62** |
|  | 495 | 0 | 675 | 0 | 870 | 205 | 5.44 | 0 | 56 | **74** |
|  | 495 | 0 | 675 | 0 | 870 | 205 | 5.44 | 0 | 90 | **83** |
|  | 272.25 | 222.75 | 675 | 0 | 870 | 205 | 5.44 | 0 | 28 | **36** |
|  | 272.25 | 222.75 | 675 | 0 | 870 | 205 | 5.44 | 0 | 56 | **66** |
|  | 272.25 | 222.75 | 675 | 0 | 870 | 205 | 5.44 | 0 | 90 | **72** |
|  | 272.25 | 222.75 | 675 | 0 | 870 | 205 | 5.44 | 14.85 | 28 | **57** |
|  | 272.25 | 222.75 | 675 | 0 | 870 | 205 | 5.44 | 14.85 | 56 | **79** |
|  | 272.25 | 222.75 | 675 | 0 | 870 | 205 | 5.44 | 14.85 | 90 | **88** |
|  | 495 | 0 | 675 | 304.5 | 565.5 | 205 | 5.44 | 0 | 28 | **59** |
|  | 495 | 0 | 675 | 304.5 | 565.5 | 205 | 5.44 | 0 | 56 | **76** |
|  | 495 | 0 | 675 | 304.5 | 565.5 | 205 | 5.44 | 0 | 90 | **82** |
|  | 272.25 | 222.75 | 675 | 304.5 | 565.5 | 205 | 5.44 | 0 | 28 | **39** |
|  | 272.25 | 222.75 | 675 | 304.5 | 565.5 | 205 | 5.44 | 0 | 56 | **64** |
|  | 272.25 | 222.75 | 675 | 304.5 | 565.5 | 205 | 5.44 | 0 | 90 | **73** |
|  | 272.25 | 222.75 | 675 | 304.5 | 565.5 | 205 | 5.44 | 14.85 | 28 | **55** |
|  | 272.25 | 222.75 | 675 | 304.5 | 565.5 | 205 | 5.44 | 14.85 | 56 | **81** |
|  | 272.25 | 222.75 | 675 | 304.5 | 565.5 | 205 | 5.44 | 14.85 | 90 | **93** |
|  | 495 | 0 | 675 | 609 | 261 | 205 | 5.44 | 0 | 28 | **58** |
|  | 495 | 0 | 675 | 609 | 261 | 205 | 5.44 | 0 | 56 | **71** |
|  | 495 | 0 | 675 | 609 | 261 | 205 | 5.44 | 0 | 90 | **76** |
|  | 272.25 | 222.75 | 675 | 609 | 261 | 205 | 5.44 | 0 | 28 | **35** |
|  | 272.25 | 222.75 | 675 | 609 | 261 | 205 | 5.44 | 0 | 56 | **59** |
|  | 272.25 | 222.75 | 675 | 609 | 261 | 205 | 5.44 | 0 | 90 | **67** |
|  | 272.25 | 222.75 | 675 | 609 | 261 | 205 | 5.44 | 14.85 | 28 | **60.5** |
|  | 272.25 | 222.75 | 675 | 609 | 261 | 205 | 5.44 | 14.85 | 56 | **68.5** |
|  | 272.25 | 222.75 | 675 | 609 | 261 | 205 | 5.44 | 14.85 | 90 | **90** |
|  | 495 | 0 | 675 | 870 | 0 | 205 | 5.44 | 0 | 28 | **49** |
|  | 495 | 0 | 675 | 870 | 0 | 205 | 5.44 | 0 | 56 | **56** |
|  | 495 | 0 | 675 | 870 | 0 | 205 | 5.44 | 0 | 90 | **66** |
|  | 272.25 | 222.75 | 675 | 870 | 0 | 205 | 5.44 | 0 | 28 | **30.5** |
|  | 272.25 | 222.75 | 675 | 870 | 0 | 205 | 5.44 | 0 | 56 | **52** |
|  | 272.25 | 222.75 | 675 | 870 | 0 | 205 | 5.44 | 0 | 90 | **55** |
|  | 272.25 | 222.75 | 675 | 870 | 0 | 205 | 5.44 | 14.85 | 28 | **51** |
|  | 272.25 | 222.75 | 675 | 870 | 0 | 205 | 5.44 | 14.85 | 56 | **56.5** |
|  | 272.25 | 222.75 | 675 | 870 | 0 | 205 | 5.44 | 14.85 | 90 | **81.5** |
|  | 410 | 0 | 825 | 0 | 1080 | 164 | 8.5 | 0 | 7 | **40** |
|  | 410 | 0 | 825 | 0 | 1080 | 164 | 8.5 | 0 | 28 | **54** |
|  | 410 | 0 | 825 | 0 | 1080 | 164 | 8.5 | 0 | 90 | **61** |
|  | 369 | 41 | 825 | 0 | 1080 | 164 | 9.5 | 0 | 7 | **42** |
|  | 369 | 41 | 825 | 0 | 1080 | 164 | 9.5 | 0 | 28 | **57.5** |
|  | 369 | 41 | 825 | 0 | 1080 | 164 | 9.5 | 0 | 90 | **66** |
|  | 328 | 82 | 825 | 0 | 1080 | 164 | 11.5 | 0 | 7 | **43.5** |
|  | 328 | 82 | 825 | 0 | 1080 | 164 | 11.5 | 0 | 28 | **59** |
|  | 328 | 82 | 825 | 0 | 1080 | 164 | 11.5 | 0 | 90 | **68** |
|  | 287 | 123 | 825 | 0 | 1080 | 164 | 12 | 0 | 7 | **38** |
|  | 287 | 123 | 825 | 0 | 1080 | 164 | 12 | 0 | 28 | **52** |
|  | 287 | 123 | 825 | 0 | 1080 | 164 | 12 | 0 | 90 | **59.5** |
|  | 410 | 0 | 825 | 540 | 540 | 176.3 | 8.5 | 0 | 7 | **37.5** |
|  | 410 | 0 | 825 | 540 | 540 | 176.3 | 8.5 | 0 | 28 | **52** |
|  | 410 | 0 | 825 | 540 | 540 | 176.3 | 8.5 | 0 | 90 | **56.5** |
|  | 369 | 41 | 825 | 540 | 540 | 176.3 | 9.5 | 0 | 7 | **40** |
|  | 369 | 41 | 825 | 540 | 540 | 176.3 | 9.5 | 0 | 28 | **53** |
|  | 369 | 41 | 825 | 540 | 540 | 176.3 | 9.5 | 0 | 90 | **60** |
|  | 328 | 82 | 825 | 540 | 540 | 176.3 | 11.5 | 0 | 7 | **41** |
|  | 328 | 82 | 825 | 540 | 540 | 176.3 | 11.5 | 0 | 28 | **53.5** |
|  | 328 | 82 | 825 | 540 | 540 | 176.3 | 11.5 | 0 | 90 | **61** |
|  | 287 | 123 | 825 | 540 | 540 | 176.3 | 12 | 0 | 7 | **36** |
|  | 287 | 123 | 825 | 540 | 540 | 176.3 | 12 | 0 | 28 | **48.5** |
|  | 287 | 123 | 825 | 540 | 540 | 176.3 | 12 | 0 | 90 | **55** |
|  | 410 | 0 | 825 | 1080 | 0 | 186 | 8.5 | 0 | 7 | **36** |
|  | 410 | 0 | 825 | 1080 | 0 | 186 | 8.5 | 0 | 28 | **45** |
|  | 410 | 0 | 825 | 1080 | 0 | 186 | 8.5 | 0 | 90 | **49** |
|  | 369 | 41 | 825 | 1080 | 0 | 186 | 9.5 | 0 | 7 | **37.5** |
|  | 369 | 41 | 825 | 1080 | 0 | 186 | 9.5 | 0 | 28 | **45.5** |
|  | 369 | 41 | 825 | 1080 | 0 | 186 | 9.5 | 0 | 90 | **49.8** |
|  | 328 | 82 | 825 | 1080 | 0 | 186 | 11.5 | 0 | 7 | **38** |
|  | 328 | 82 | 825 | 1080 | 0 | 186 | 11.5 | 0 | 28 | **47** |
|  | 328 | 82 | 825 | 1080 | 0 | 186 | 11.5 | 0 | 90 | **49.8** |
|  | 287 | 123 | 825 | 1080 | 0 | 186 | 12 | 0 | 7 | **33** |
|  | 287 | 123 | 825 | 1080 | 0 | 186 | 12 | 0 | 28 | **43** |
|  | 287 | 123 | 825 | 1080 | 0 | 186 | 12 | 0 | 90 | **46** |
